# Ultrasound-Assisted Ionic Liquid Extraction, Macroporous Resin Purification, and Hypoglycemic Effects of Total Flavonoids from *Polygonum perfoliatum* L.

**DOI:** 10.3390/molecules31162758

**Published:** 2026-08-08

**Authors:** Yanping Zhang, Haichao Zuo, Chenxi Zhang, Di Gao, Yibo Wen, Xinxin Jiang

**Affiliations:** Chemistry and Chemical Engineering School, Henan University of Science and Technology, Luoyang 471023, China

**Keywords:** *Polygonum perfoliatum* L., total flavonoids, extraction and purification, ionic liquid, hypoglycemic Effects, type 2 diabetes mellitus

## Abstract

In this study, total flavonoids from *Polygonum perfoliatum* L. (PPTF) were extracted using ionic liquid-assisted ultrasound and purified by macroporous resin. After optimization, the total flavonoid content increased from 33.9% to 71.2%. HPLC identified hyperoside, kaempferol-3-O-rutinoside, and quercetin. In vitro experiments showed that PPTF exhibited good free radical scavenging activity and inhibitory effects on α-glucosidase and α-amylase. Within the concentration range of 3.13–12.50 μg/mL, PPTF was non-toxic to HepG2 cells and improved glucose uptake, promoted glycogen synthesis, and reduced lipid accumulation in insulin-resistant cells in a dose-dependent manner. In a type 2 diabetic mouse model induced by high-fat diet combined with streptozotocin, intragastric administration of PPTF for four weeks significantly lowered fasting blood glucose in the high-dose group, improved oral glucose tolerance and insulin sensitivity, increased hepatic and muscle glycogen, normalized serum lipid profiles, and alleviated pathological damage in the liver and pancreas. PPTF exhibits favorable hypoglycemic and lipid-regulating effects in both cellular and animal models, demonstrating its potential for development as an adjunctive functional product for glycemic management.

## 1. Introduction

*Polygonum perfoliatum* L. known in traditional Chinese medicine as “Gangbangui” and also referred to by the vernacular names “She Daotui” and “He Baicao”, is the dried aerial part of this medicinal plant [1]. It is widely distributed across China, occurring in most regions including the Northeast, North, East, South, and Southwest. Its main production areas include Zhejiang, Shandong, Jiangxi, Fujian, Guangdong, Guangxi, Sichuan, Hunan, and Guizhou provinces [2]. Modern pharmacological studies have demonstrated that *Polygonum perfoliatum* L. possesses broad and significant pharmacological effects, including anti-inflammatory, antioxidant, antitumor, and antiviral activities [3,4,5]. For instance, Fan et al. isolated quercetin-3-O-β-D-glucuronide from this plant and confirmed its notable anti-inflammatory and anti-influenza virus activities, and further established an HPLC method for its quantitative analysis [6]. In support of these findings, a recent qualitative analysis of the chemical constituents of *P. perfoliatum* has systematically characterized its complex phytochemical profile and simultaneously evaluated its associated biological activities, further reinforcing the medicinal value of this plant [7]. To date, a variety of chemical components have been isolated and identified from this plant, including flavonoids, phenolic acids, terpenoids, anthraquinones, phenylpropanoids, and polysaccharides. Among these, flavonoids are the most abundant and diverse group, constituting the major part of the chemical basis of *Polygonum perfoliatum* L. [8].

Traditional methods for flavonoid extraction, including reflux, maceration, and Soxhlet extraction, are simple to operate and require low equipment investment; however, they generally exhibit drawbacks such as high solvent usage, long extraction duration, and susceptibility to degradation of heat-sensitive constituents [9,10]. In the case of *P. perfoliatum*, previous studies have already applied conventional extraction and purification techniques to obtain its flavonoid fraction and confirmed its antiviral and antioxidant activities. Nevertheless, these traditional methods still suffer from the aforementioned limitations, necessitating the development of more efficient and greener extraction strategies [11]. Ionic liquids are low-temperature molten salts consisting of organic cations and inorganic/organic anions. They exhibit extremely low vapor pressure, high thermal stability, non-flammability, and outstanding dissolving ability for both polar and non-polar compounds. Moreover, their cation/anion structures can be directionally adjusted, earning them the reputation of “designer solvents” [12]. Imidazolium-based ionic liquids are the most commonly employed class for flavonoid extraction. For instance, 1-butyl-3-methylimidazolium bromide ([Bmim]Br) forms strong hydrogen bonds with cellulose and lignin in plant cell walls, effectively disrupting the cell wall and promoting the release of intracellular flavonoids into the extraction solvent. Scanning electron microscopy has verified that, under ultrasonic assistance, [Bmim]Br effectively ruptures the cell wall and enhances flavonoid dissolution [13]. The coupling of ultrasound-assisted extraction with ionic liquids has emerged as a research focus. The cavitation effect of ultrasound potentiates the cell wall disruption by ionic liquids, and their synergistic action markedly reduces extraction time and increases flavonoid yield.

Flavonoids possess significant antioxidant and hypoglycemic activities [14,15,16]. The in vitro antioxidant activity of flavonoids is commonly evaluated through a series of chemical chromogenic reactions [17]. Flavonoids are capable of activating the insulin signaling pathway, consequently enhancing glucose uptake, modulating glucolipid metabolism, and improving insulin resistance [18]. The in vitro assessment of hypoglycemic effects generally involves two approaches. The first is the measurement of inhibitory activities against α-glucosidase and α-amylase, which serves to evaluate the potential for retarding postprandial hyperglycemia [19]. The second is based on an insulin-resistant HepG2 cell model to examine the effects of drugs on glucose consumption, glucose uptake, and signaling pathway proteins [20,21]. For in vivo evaluation, a type 2 diabetic mouse model induced by a high-fat diet combined with streptozotocin (STZ) is commonly used [22]. The hypoglycemic effect is comprehensively assessed by measuring parameters such as fasting blood glucose, glucose tolerance, insulin tolerance, and blood lipid levels [23]. Studies have shown that flavonoids can significantly reduce fasting blood glucose, improve the serum lipid profile, and alleviate histopathological damage. The underlying mechanisms are associated with restoration of IRS phosphorylation, activation of the PI3K/Akt signaling pathway, promotion of AMPK phosphorylation, and translocation of GLUT4 to the cell membrane [24,25]. These in vitro and in vivo evaluation methods together constitute a complete system for studying the hypoglycemic activity of flavonoids.

Type 2 diabetes mellitus (T2DM) has become a global public health challenge, with its prevalence continuing to rise. In 2022, there were 828 million adults with diabetes worldwide [26]. In China, the prevalence of diabetes increased from 2.5% in 1994 to 13.0% in 2021, affecting over 140 million patients, yet the rate of treatment target attainment remains relatively low [27]. Traditional Chinese medicine (TCM) has accumulated extensive experience in blood glucose control. Studies have shown that TCM also exhibits favorable hypoglycemic effects, with fewer adverse reactions and the advantage of multi-target actions [28]. For instance, the intervention of traditional Chinese medicine in T2DM involves multi-level mechanisms, including promoting glucose transport, improving glycogen metabolism, stimulating GLP-1 secretion, protecting pancreatic islets, and regulating gut microbiota [29]. Furthermore, the co-administration of traditional Chinese medicine and Western medicine may elicit synergistic effects. For instance, the use of Liuwei Dihuang Wan in conjunction with gliclazide results in a more rapid therapeutic effect and reduced adverse reactions [30]. Currently, conventional Western drugs remain inadequate for addressing individualized requirements. Nearly half of all patients fail to reach the desired glycated hemoglobin target, and the use of these medications is accompanied by side effects including gastrointestinal disturbances, hypoglycemia, and weight gain [31,32]. Therefore, exploring natural products with multi-target regulatory effects and high safety has become an important direction in diabetes drug research and development [33].

Currently, studies on the hypoglycemic activity of *Polygonum perfoliatum* L. are relatively scattered and limited in number. Additionally, although total flavonoids from certain *Polygonum* plants have demonstrated hepatoprotective properties, whether the total flavonoids from *P. perfoliatum* (PPTF) possess both hypoglycemic and hepatic protective actions in the context of diabetic complications remains to be elucidated [34]. Therefore, this study aims to employ ultrasound-assisted ionic liquid extraction combined with macroporous resin purification to obtain PPTF, establish a reliable HPLC method for flavonoid content determination, and comprehensively evaluate its anti-type 2 diabetes activity through both in vitro and in vivo experiments. This research will provide a scientific basis for the deep development of this traditional Chinese medicinal resource and the preparation of adjuvant hypoglycemic functional products, and also serve as a reference for discovering bioactive components relevant to glycemic control.

## 2. Results and Discussion

### 2.1. Experimental Results of PPTF Extraction

#### 2.1.1. Rutin Standard Curve

**Figure 1:**
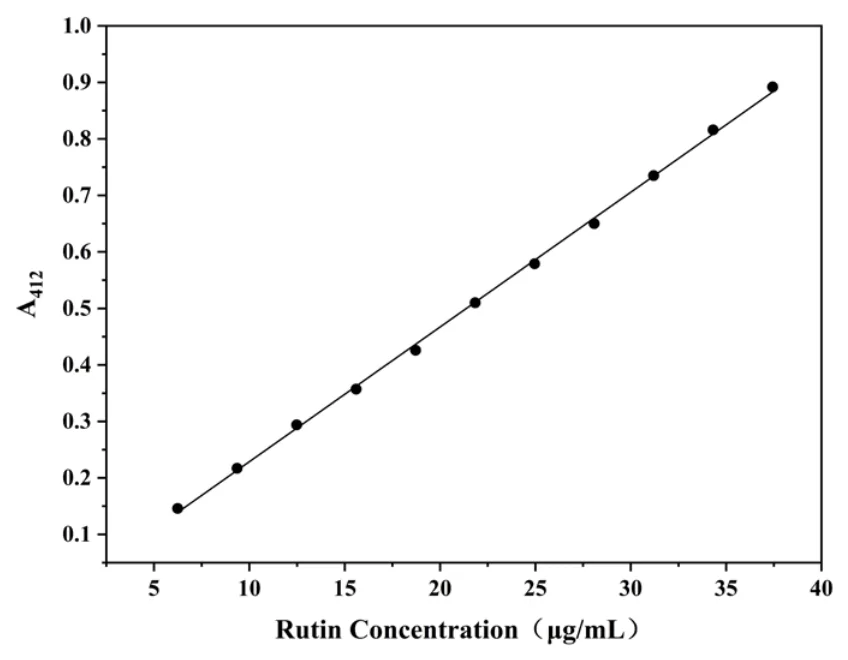
Standard curve of rutin concentration.

The absorbance of rutin reference standards at different concentrations was measured, and the standard calibration curve was obtained with the regression equation: y = 0.0239x − 0.01 (R^2^ = 0.9992), indicating good linearity within the concentration range of 6.24–37.44 μg/mL (Figure 1).

#### 2.1.2. Results of Ionic Liquid Screening and Single-Factor Experiments

**Figure 2:**
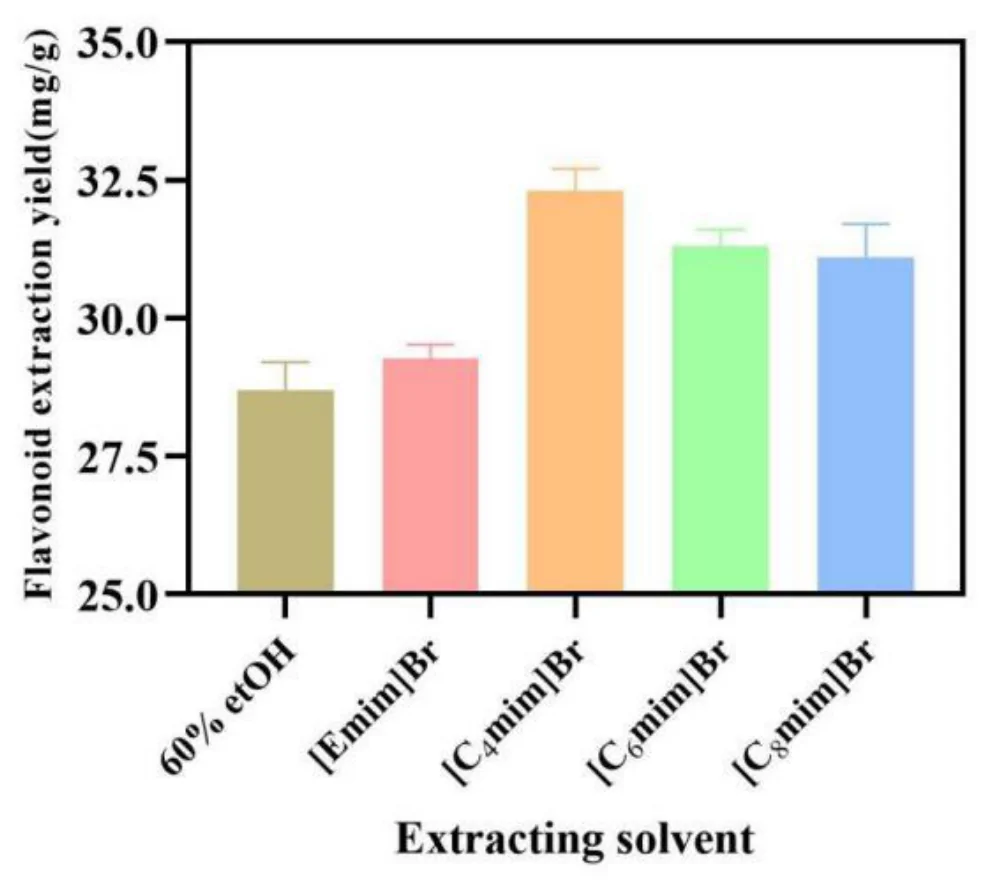
Ionic liquid screening results. Data are presented as mean ± SD (*n* = 3).

As shown in Figure 2, in the ionic liquid screening experiment, compared with the condition using only 60% (*v*/*v*) ethanol, the addition of each of the four ionic liquids increased the extraction yield of PPTF. Among them, [C_4_mim]Br provided the highest improvement in flavonoid yield; therefore, it was selected for subsequent single-factor experiments.

**Figure 3:**
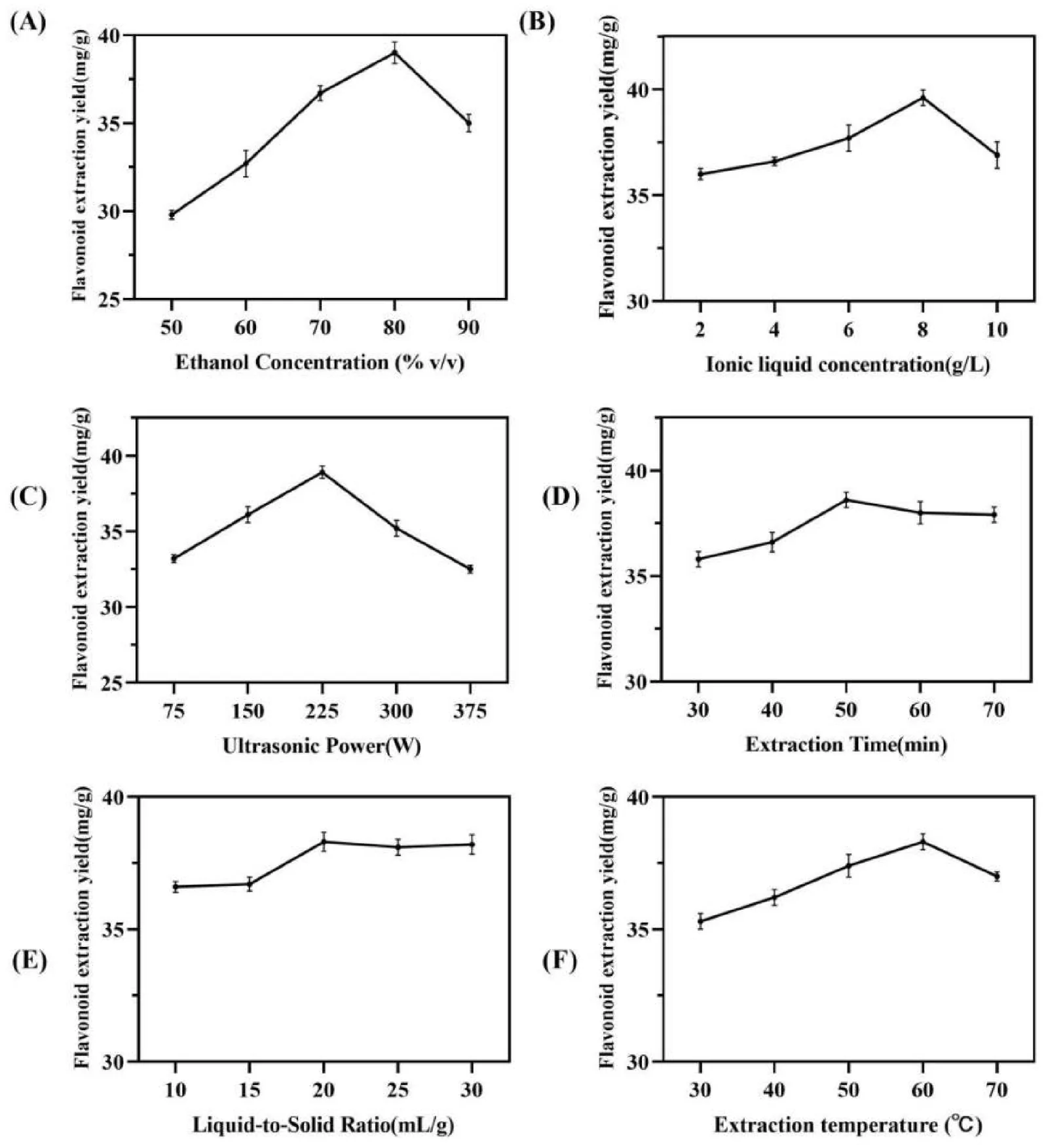
Results of single-factor experiment. (**A**) Ethanol volume fraction; (**B**) Ionic liquid concentration; (**C**) Ultrasonic power (**D**) Extraction time; (**E**) Solid-to-liquid ratio; (**F**) Extraction temperature. Data are presented as mean ± SD (*n* = 3).

The single-factor experimental results (Figure 3) indicated that as the ethanol volume fraction increased from 50% to 80%, the extraction yield of total flavonoids rose accordingly, reaching its maximum at 80%; further increasing to 90% led to a decrease, which is closely related to the polarity and dissolution behavior of flavonoid compounds [35]. The extraction yield exhibited a characteristic parabolic response to increasing ionic liquid concentration, reaching an optimum at an intermediate concentration before declining. This decline is mechanistically attributed to the increased viscosity of the extraction solution at higher concentrations, which inhibits the cavitation effect of ultrasound and elevates mass transfer resistance [36]. The extraction yield increased with ultrasonic power up to 225 W, and then decreased with further increase; excessively strong cavitation might generate localized high temperature and high pressure, potentially leading to the degradation of flavonoids [37]. As extraction time was prolonged from 30 min to 50 min, the yield increased to 38.6 mg/g, and then slightly decreased with further extension, which could be explained by the saturation of flavonoid dissolution and decomposition caused by prolonged ultrasonic treatment. The solid-to-liquid ratio had no significant effect on the extraction yield; a ratio of 1:20 (g/mL) gave a slightly better result, and the yield tended to stabilize with further increase in solvent volume. The extraction temperature gave the highest yield (38.3 mg/g) at 60 °C, while the yield declined above 70 °C due to oxidation, polymerization, or degradation of flavonoids under high temperature [38].

#### 2.1.3. Results of Optimization of PPTF Extraction Conditions by Response Surface Methodology

In this study, three key independent variables—ethanol volume fraction (A), ionic liquid concentration (B), and ultrasonic power (C)—were selected, and the extraction yield of PPTF was used as the response variable. A total of 17 experimental runs were designed and conducted using Box–Behnken design, and the observed response values are presented in Table 1.

Based on the response surface results, a quadratic polynomial regression model for PPTF extraction yield was established as follows:

Extraction yield (mg/g) = −59.05+ 1.1375A + 8.95625B + 0.152167C − 0.015AB − 0.0002AC − 0.001833BC − 0.06125A^2^ − 0.44687B^2^ − 0.000273C^2^.

In coded-variable form, the equation is:

Extraction yield (mg/g) = 39.65 + 0.075A + 0.3875B + 0.1125C − 0.3AB − 0.15AC − 0.275BC − 0.6125A^2^ − 1.825B^2^ − 1.575C^2^

Analysis of variance (ANOVA) for the regression model is presented in Table 2. The model F-value of 129.2 with a *p*-value < 0.0001 confirmed the high significance of the model, while the lack-of-fit *p*-value of 0.1883 indicated no significant lack of fit (*p* > 0.05), supporting the model’s reliability for predicting extraction yields. Among the linear terms, ionic liquid concentration (B) exerted a highly significant effect (*p* = 0.0002), whereas ethanol volume fraction (A) and ultrasonic power (C) showed no significant linear effects (*p* > 0.05). For the interaction terms, AB (*p* = 0.007) and BC (*p* = 0.0106) were significant, suggesting a synergistic effect between ionic liquid concentration and the other two factors. All quadratic terms (A^2^, B^2^, C^2^) were highly significant (*p* < 0.0001), confirming a parabolic, nonlinear relationship between the three factors and extraction yield. This observation is consistent with the single-factor results, where either excessively high or low factor levels reduced extraction efficiency. The model predicted the optimal extraction conditions as follows: ethanol volume fraction of 79.1%, ionic liquid concentration of 8.2 g/L, and ultrasonic power of 222 W, with a predicted extraction yield of 39.73 mg/g. Considering the single-factor results and practical operability, the combined optimal conditions were adjusted to ethanol volume fraction 79%, ionic liquid concentration 8 g/L, ultrasonic power 220 W, extraction time 50 min, solid-to-liquid ratio 1:20, and extraction temperature 60 °C. Under these adjusted conditions, the actual measured extraction yield was 39.51 mg/g, which closely matched the predicted value (39.73 mg/g), thereby confirming the reliability of the model.

**Figure 4:**
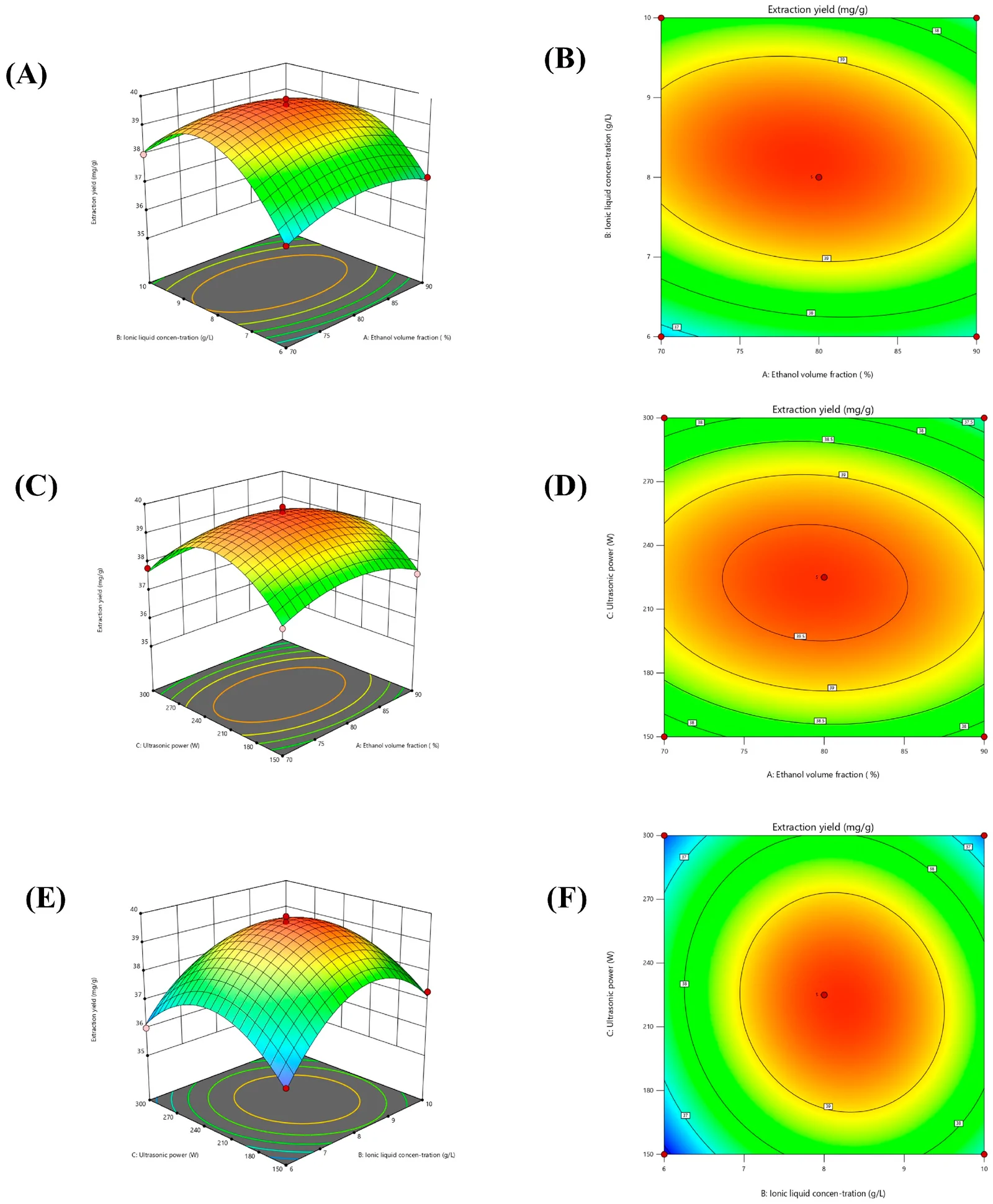
Response surface and contour plots of influencing factors on extraction yield. (**A**,**B**) Ethanol volume fraction and ionic liquid concentration; (**C**,**D**) Ethanol volume fraction versus ultrasonic power; (**E**,**F**) Ionic liquid concentration versus ultrasonic power. The color gradient represents the extraction yield (mg/g), with red indicating higher values and blue indicating lower values.

Based on the response values obtained from the Box–Behnken design and the ANOVA results, contour plots and response surface plots (Figure 4) were generated to visualize the interactive effects of the three factors on the extraction yield of PPTF.

The interactive effects of the experimental variables on extraction yield were visualized using 3D response surface and 2D contour plots (Figure 4). The steepness of the surface and the shape of the contours indicate the significance of the interactions. A strong synergistic interaction was identified between ethanol volume fraction and ionic liquid concentration (Figure 4A,B). The steep slope along the ionic liquid concentration axis demonstrates its dominant role in enhancing the extraction yield. The elliptical contours confirm the significant interaction between these two factors. In contrast, the nearly circular contours for the interaction between ethanol volume fraction and ultrasonic power (Figure 4C,D) indicate that this interaction is not statistically significant. Notably, a significant interaction was observed between ionic liquid concentration and ultrasonic power, characterized by a highly curved surface and elliptical contours (Figure 4E,F). This suggests a trade-off relationship: a high extraction yield can be maintained either by increasing the ionic liquid concentration or by intensifying the ultrasonic power. For instance, at low ionic liquid concentrations, a higher ultrasonic power is required to achieve sufficient cell wall disruption, whereas at high concentrations, the potent solvent effect allows for a reduction in power input. This observation aligns perfectly with the significant BC interaction term revealed by ANOVA.

The ultrasound-assisted ionic liquid extraction method established in this study offers several advantages: high extraction yield short extraction time, low solvent consumption, and the use of ionic liquids as green solvents. The synergistic effect between ultrasound cavitation and ionic liquid-mediated cell wall disruption not only improves extraction efficiency but also effectively preserves the stability of heat-sensitive flavonoid components.

### 2.2. Purification Results of PPTF on HPD-100 Macroporous Resin

#### 2.2.1. Results of the Breakthrough Curve for Dynamic Adsorption of Flavonoids

**Figure 5:**
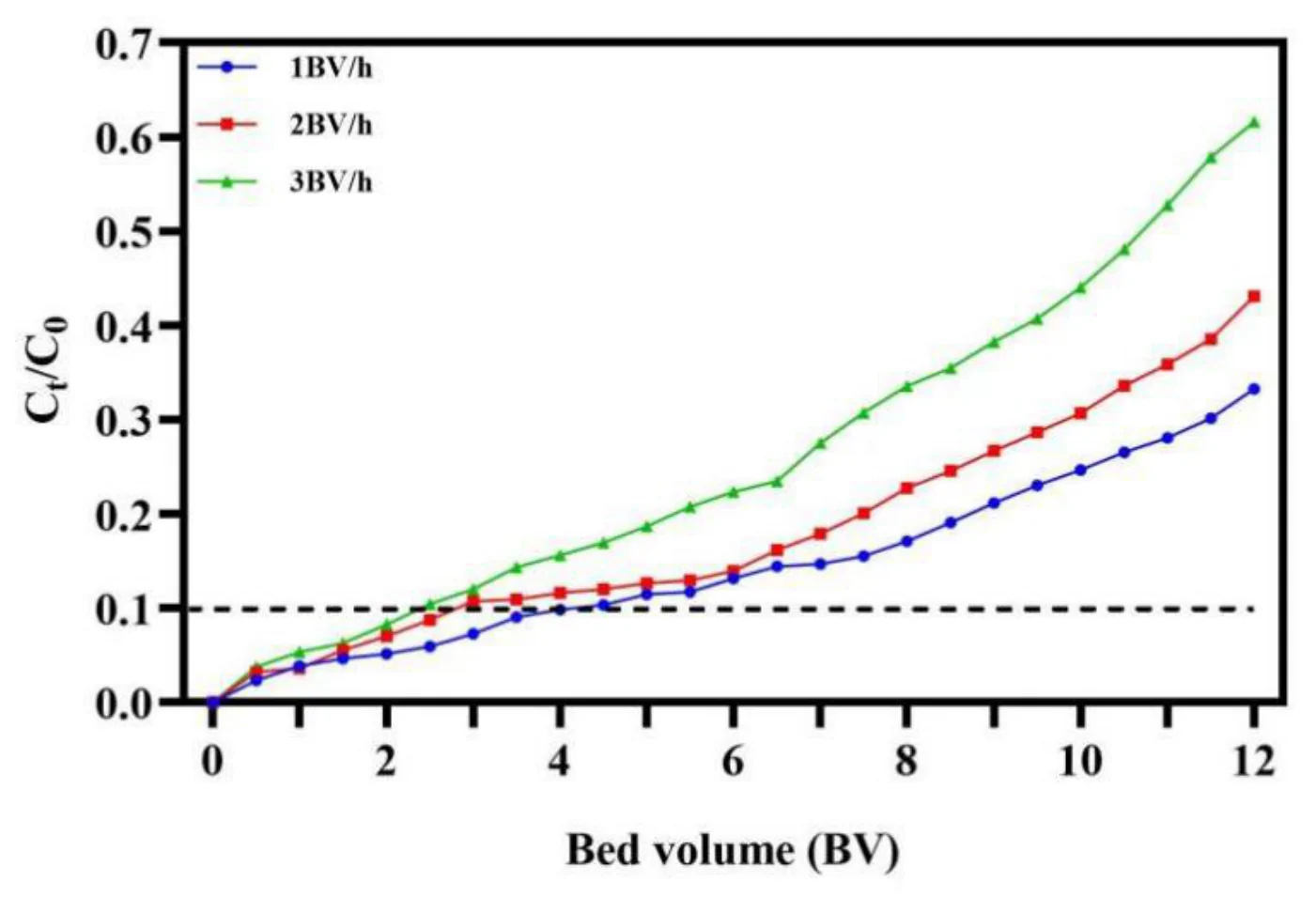
Breakthrough curve for dynamic adsorption of flavonoids on HPD-100 resin. Data are presented as mean ± SD (*n* = 3).

The dynamic adsorption breakthrough curves are presented in Figure 5. At a flow rate of 1 BV/h, the effluent concentration increased slowly with the loaded volume and first exceeded the breakthrough point at 4.5 BV. When the flow rate was increased to 2 BV/h, mass transfer accelerated, and breakthrough occurred earlier, at 3 BV. Further increasing the flow rate to 3 BV/h advanced the breakthrough point to 2.5 BV. These results indicate that the flow rate significantly affects the breakthrough point: a higher flow rate shortens the residence time of flavonoids in the column, preventing sufficient diffusion into the resin pores and leading to earlier breakthrough, thereby reducing the effective adsorption capacity [39]. The flow rate of 1 BV/h gave the highest resin utilization but a longer operation cycle, whereas 3 BV/h offered higher productivity at the cost of considerable adsorption capacity loss. Considering both adsorption efficiency and process time, 2 BV/h was selected as the optimal flow rate for dynamic adsorption, with a breakthrough point of approximately 2.8 BV, which provides a good balance between adsorption capacity and operational efficiency.

#### 2.2.2. Results of the Gradient Elution Curve of Flavonoids

**Figure 6:**
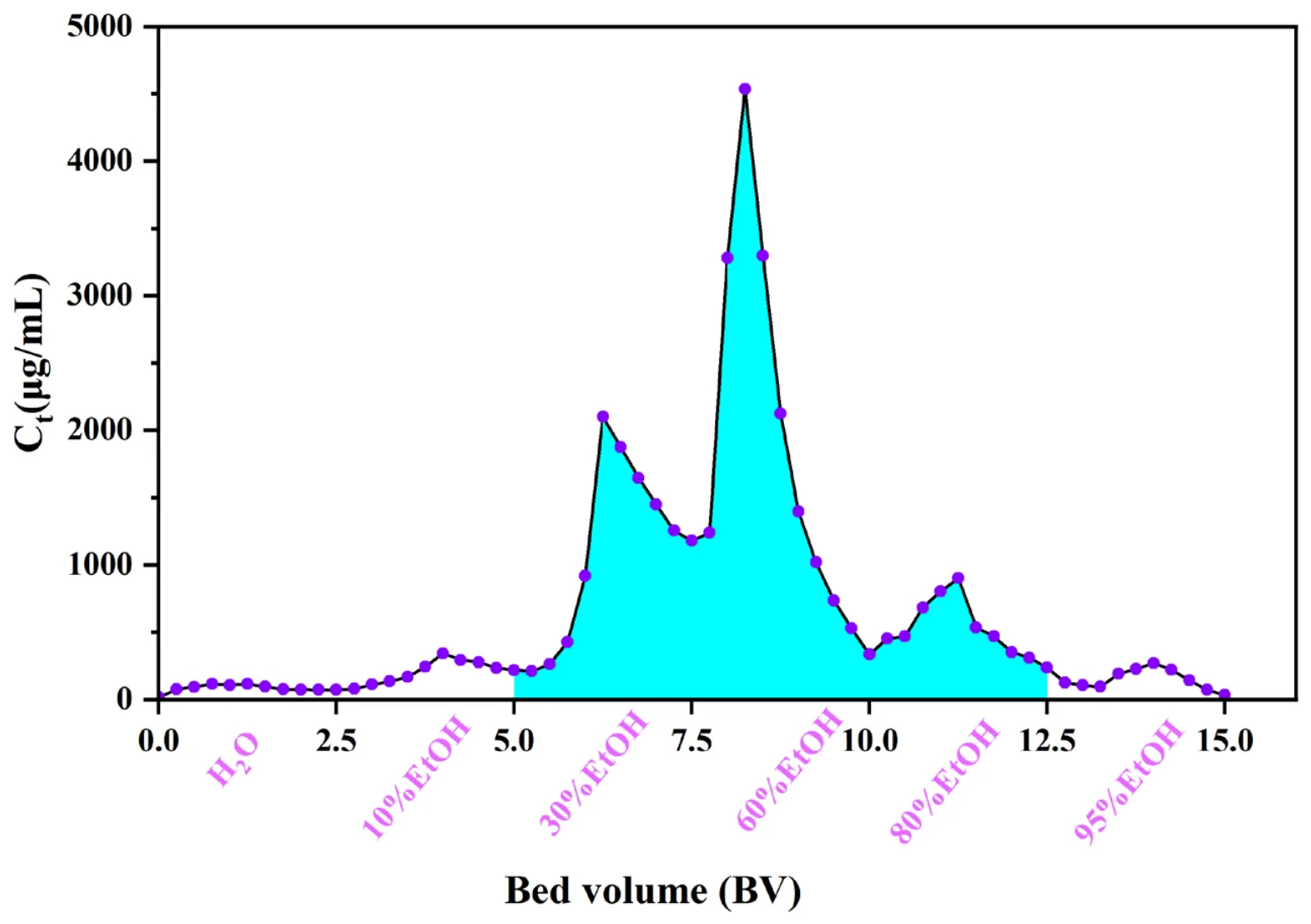
Gradient elution curve of flavonoids on HPD-100 resin. Data are presented as mean ± SD (*n* = 3).

The gradient elution curve is shown in Figure 6. In the water elution stage, the flavonoid concentration was low, indicating that water mainly removed strongly polar impurities such as polysaccharides and proteins, while flavonoids were barely eluted [40]. In the 10% ethanol stage, the concentration increased slightly, suggesting that a small amount of highly polar flavonoid glycosides was eluted. In the 30% ethanol stage, the concentration continued to rise, indicating the desorption of moderately polar flavonoid glycosides. The flavonoid concentration reached its highest value in the 60% ethanol stage, demonstrating that most flavonoid components were effectively eluted at this step. In the 80% and 95% ethanol stages, the concentration dropped sharply, indicating that the remaining flavonoids were almost completely eluted. During the gradient elution process, the water and low-concentration ethanol elution stages could remove most of the ionic liquid, and HPD-100 resin exhibited negligible adsorption affinity for imidazolium-based ionic liquids; therefore, the residual ionic liquid in the purified flavonoid samples could be considered negligible. After the entire purification process, the PPTF extract increased from 33.9% to 71.2%, demonstrating that this purification procedure significantly improved the purity of flavonoids.

It should be noted that HPD-100 resin exhibits negligible adsorption affinity for imidazolium-based ionic liquids such as [C_4_mim]Br. As a non-polar macroporous adsorption resin, HPD-100 primarily adsorbs target compounds through van der Waals forces and hydrophobic interactions, which are effective for non-polar or weakly polar organic molecules such as flavonoids but provide insufficient driving force for ionic compounds. Previous studies have demonstrated that macroporous resins can effectively separate target analytes from [C_4_mim]Br solutions while allowing the ionic liquid to remain in the effluent [41,42]. Systematic investigations on the adsorption behavior of imidazolium-based ionic liquids onto various resins further confirmed that resins without functional groups exhibit poor adsorption capacity for both hydrophilic and hydrophobic ionic liquids [43]. Moreover, studies on the recycling of ionic liquids and regeneration of HPD100B resin have shown that the ionic liquid can be recovered from the solution phase after resin treatment, indicating that it is not retained by the resin matrix. Therefore, during the gradient elution process, the water and low-concentration ethanol elution stages effectively remove most of the ionic liquid, and the residual ionic liquid in the purified flavonoid samples can be considered negligible.

#### 2.2.3. Results of HPLC Identification of Flavonoid Components and Their Contents Before and After Purification

**Figure 7:**
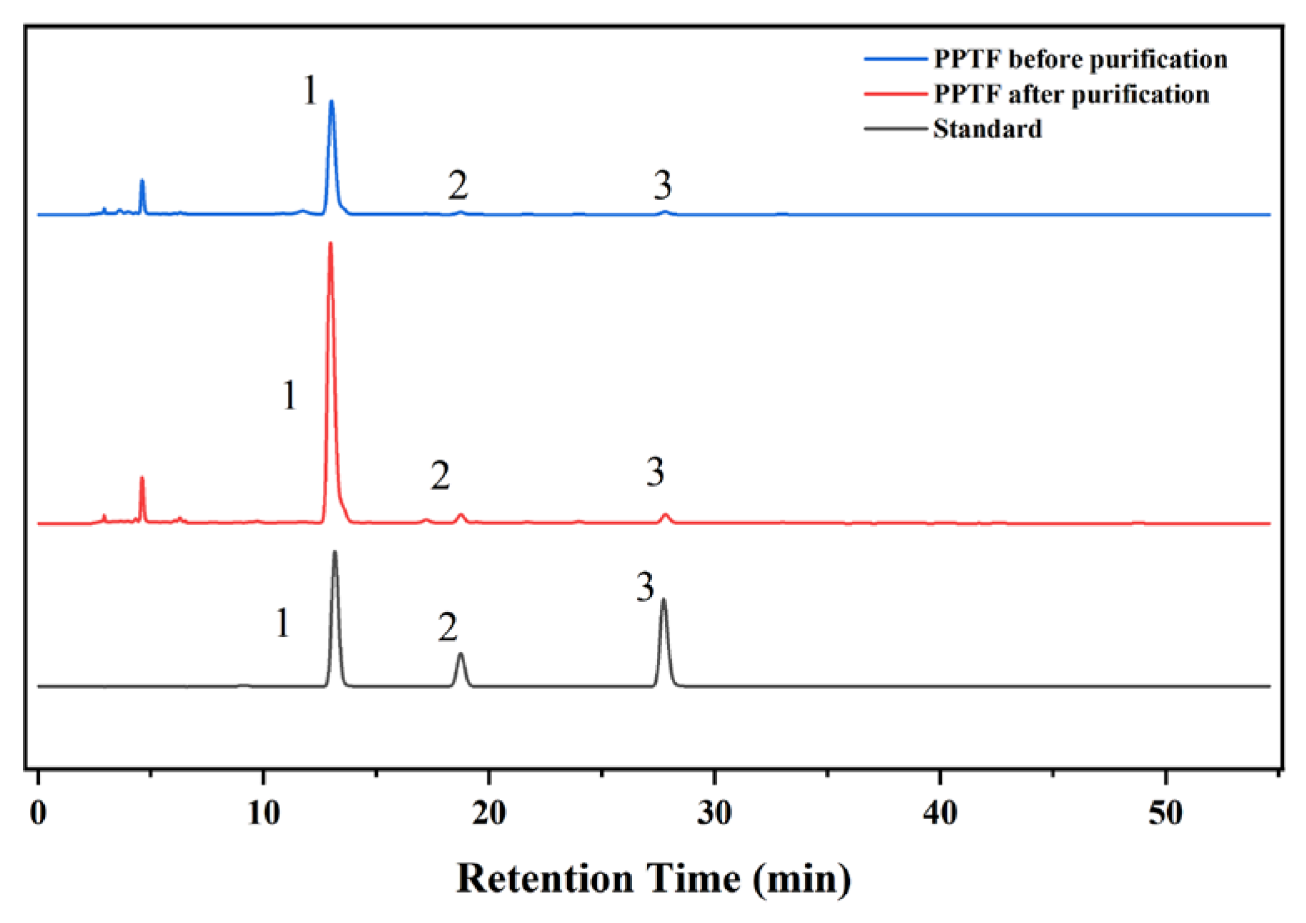
HPLC chromatogram of PPTF (1: Hyperoside, 2: Kaempferol-3-O-rutinoside, 3: Quercetin). Data are presented as mean ± SD (*n* = 6).

The HPLC chromatogram of flavonoids from PPTF is shown in Figure 7. The regression equations for the three flavonoid components were as follows: hyperoside, y = 25.756x + 125.37 (R^2^ = 0.9998), linear range 42.58–681.20 μg/mL; kaempferol-3-O-rutinoside, y = 14.711x + 36.247 (R^2^ = 0.9993), linear range 20.70–331.20 μg/mL; quercetin, y = 26.143x − 73.011 (R^2^ = 0.9993), linear range 31.40–502.40 μg/mL. Each component exhibited good linearity within its respective concentration range. Method validation results (Table 3) showed that the average recoveries of hyperoside, kaempferol-3-O-rutinoside, and quercetin were 98.59%, 99.23%, and 98.72%, respectively. The relative standard deviations (RSDs) for stability within 24 h were 1.42%, 1.13%, and 0.94%, and those for repeatability (five parallel measurements) were 1.34%, 1.61%, and 1.27%, all below 2%. These results indicate that the established HPLC method possesses good linearity, accuracy, stability, and repeatability, and is suitable for the determination of PPTF. Table 4 shows the flavonoid contents before and after purification, determined from lyophilized powder solutions at the same concentration (100 mg/mL). The flavonoid profile in this study differs from our previous work, in which quercetin was identified as the major flavonoid regardless of geographical origin, whereas hyperoside appeared as the dominant compound in the present study. This discrepancy can be attributed to several factors: (1) different detection wavelengths (360 nm vs. 320 nm), which affect the relative response of individual flavonoids; (2) different sample types (purified fractions vs. crude extracts), as the purification process may alter the relative abundance of components; and (3) different analytical objectives (marker quantification vs. comprehensive profiling).

It should be noted that the flavonoid profile observed in this study differs from that reported in our previous work, in which quercetin was identified as the major flavonoid regardless of geographical origin, whereas hyperoside appeared as the dominant compound in the present study. This discrepancy can be attributed to two main factors. First and foremost, geographical origin plays a critical role. The plant material used in this study was collected from a single location (Jieyang, Guangdong), whereas in our previous study, we analyzed samples from 11 different geographical origins, and the reported flavonoid profile represented the overall average or composite result across these origins. It is well established that the flavonoid composition of medicinal plants can vary significantly depending on environmental factors such as soil composition, climate, altitude, and harvest time. Therefore, the simplified profile observed in this study may reflect the specific chemotype of that particular origin rather than the average profile across multiple origins. Second, sample type also contributes to the observed differences: the present study analyzed purified total flavonoid fractions after macroporous resin purification, whereas the previous study analyzed crude extracts. The purification process may selectively enrich or deplete certain flavonoids, altering the relative abundance. Thus, the observed shift from quercetin to hyperoside as the dominant compound is likely a combined result of both geographical variation and the purification process.

The data in Table 3 and Table 4 reflect two fundamentally different experimental processes. Table 3 presents the spike recovery experiment, in which purified reference flavonoids were added to crude extract samples and then subjected to the complete purification procedure. This experiment was designed to evaluate the accuracy and precision of the method, specifically to determine whether the purification steps cause significant loss of the target compounds. The recovery rates exceeded 96%, indicating that the purification process did not result in appreciable loss of the three target flavonoids. Table 4, in contrast, compares the absolute contents of these three flavonoids in the crude extract and the purified PPTF, with concentration ratios ranging from 2.49 to 2.88, reflecting the enrichment efficiency of the purification process for these three flavonoids. It should be noted that recovery and enrichment factor represent distinct concepts: recovery merely indicates negligible loss of target compounds during the purification steps, whereas the enrichment factor depends not only on recovery but also on the extent of selective removal of non-flavonoid components (such as sugars, proteins, pigments, etc.) and the mass yield of PPTF relative to the starting crude extract.

The sum of the three quantified flavonoids in Table 4 is approximately 1.6 mg/100 mg PPTF (1.6%), substantially lower than the 71.2% total flavonoid content determined by the colorimetric method. This discrepancy primarily results from the different selectivity of the two analytical methods: the colorimetric method, based on aluminum ion–flavonoid complexation, responds broadly to flavonols, flavones, flavanones, and even some phenolic acids, whereas the HPLC method quantifies only three specific components (hyperoside, kaempferol-3-O-rutinoside, and quercetin). The remaining approximately 70% of the colorimetric signal likely originates from numerous other flavonoid compounds present in PPTF at lower individual concentrations. Regarding the absence of prominent additional peaks in the HPLC chromatogram, a probable explanation is that many flavonoids may co-elute under the current conditions or appear as small, broad peaks difficult to distinguish from the baseline, reflecting the resolution and sensitivity limitations of the HPLC-UV method. Furthermore, it is well documented that this colorimetric method tends to overestimate total flavonoid content in complex plant extracts due to interference from non-flavonoid phenolic compounds and the non-specificity of the complexation reaction [44,45]. Thus, the substantial gap between 1.6% and 71.2% results from the combined effects of the fundamental differences in detection principles and the inherent overestimation tendency of the colorimetric method.

### 2.3. Results of In Vitro Activity Evaluation of PPTF

#### 2.3.1. Results of In Vitro Antioxidant Activity of PPTF

**Figure 8:**
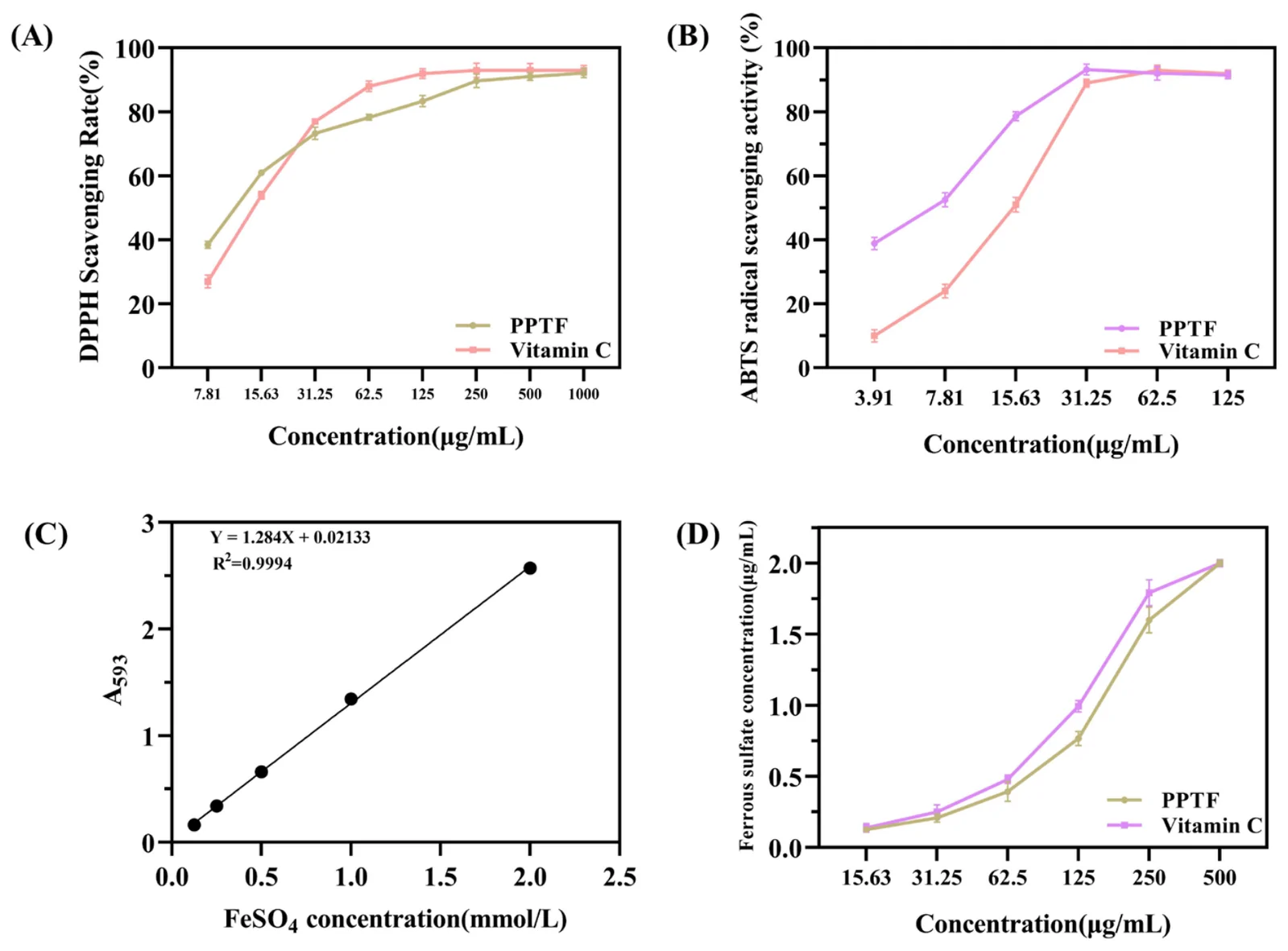
In vitro antioxidant activity of PPTF. (**A**) DPPH; (**B**) ABTS; (**C**) Standard curve of ferrous sulfate; (**D**) FRAP. Data are presented as mean ± SD (*n* = 6).

The DPPH radical scavenging assay (Figure 8A) showed that PPTF scavenged DPPH radicals in a concentration-dependent manner. PPTF exhibited concentration-dependent free radical scavenging activities across all three assays. Notably, in the ABTS assay, PPTF demonstrated superior activity compared to VC at lower concentrations, highlighting its potent capacity to scavenge physiologically relevant free radicals. This differential performance between DPPH and ABTS assays likely reflects the diversity of flavonoid species in PPTF, each with distinct redox potentials and radical selectivity. The ABTS radical scavenging assay (Figure 8B) demonstrated that PPTF exhibited good antioxidant activity. At low concentrations, the scavenging rate of PPTF was significantly higher than that of VC. The IC_50_ values of PPTF for ABTS and DPPH radicals were determined to be 7.10 μg/mL and 11.80 μg/mL, respectively, indicating that PPTF possesses strong radical scavenging capacity. The standard curve for ferrous sulfate was y = 1.2837x + 0.0213 (R^2^ = 0.9994), with a linear range of 0.25–2 mmol/L. The FRAP assay (Figure 8D) showed that the reducing power of PPTF increased with concentration in a dose-dependent manner. Within the concentration range of 15.63–125.00 μg/mL, the FeSO_4_ equivalent of VC was slightly higher than that of PPTF; at 250.00 μg/mL, the gap narrowed. Overall, PPTF exhibited good total antioxidant capacity.

#### 2.3.2. Results of In Vitro Enzyme Inhibitory Activity of PPTF

**Figure 9:**
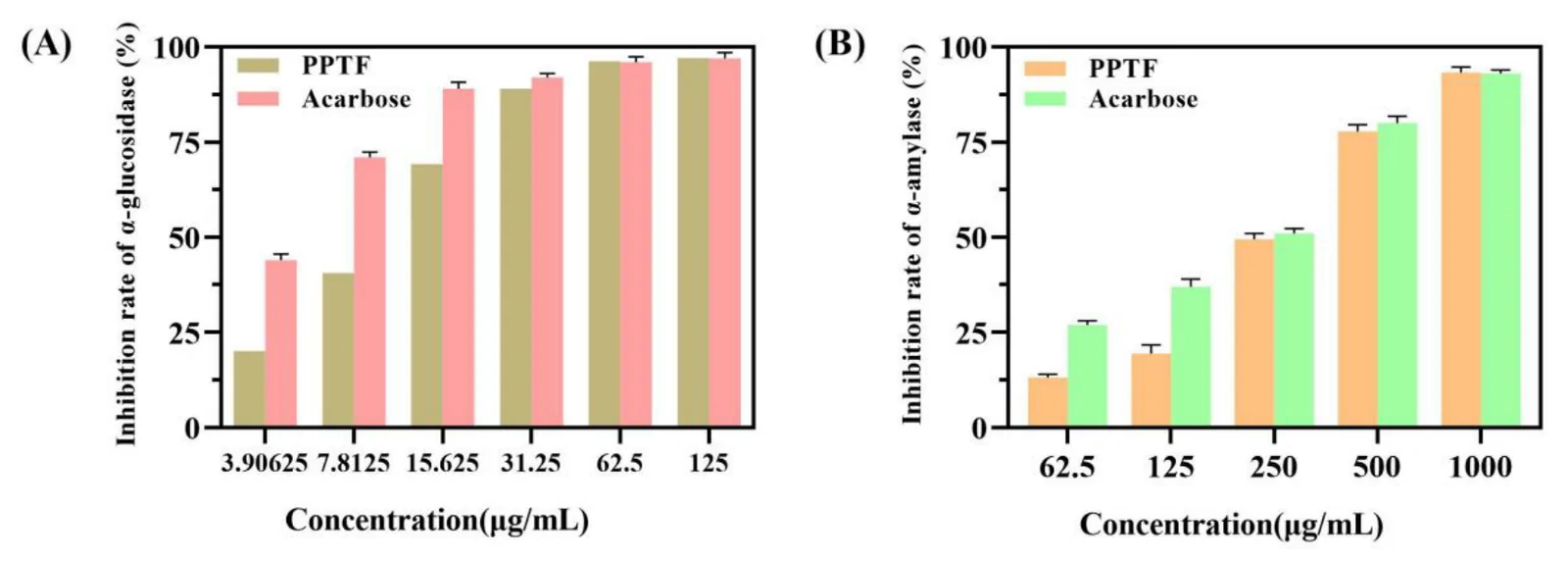
In vitro enzyme inhibitory activity of PPTF. (**A**) α-Glucosidase inhibitory activity; (**B**) α-Amylase inhibitory activity. Data are presented as mean ± SD (*n* = 6).

The α-glucosidase inhibition assay results (Figure 9A) showed that PPTF inhibited the enzyme in a dose-dependent manner, although its overall activity was weaker than that of the positive control acarbose. PPTF showed a notable preference for inhibiting α-glucosidase over α-amylase. The IC_50_ values of PPTF against α-glucosidase and α-amylase were determined to be 11.00 μg/mL and 250.00 μg/mL, respectively, indicating that PPTF is a more potent inhibitor of α-glucosidase than of α-amylase. This selective inhibition profile is pharmacologically advantageous: while α-glucosidase blockade effectively attenuates postprandial glycemic spikes, excessive α-amylase inhibition is associated with undigested carbohydrate fermentation in the colon, leading to gastrointestinal discomfort—a common side effect of non-selective inhibitors such as acarbose. Therefore, the selectivity of PPTF suggests a safer profile for long-term glycemic management. This selective inhibition profile is beneficial for controlling postprandial blood glucose while reducing the gastrointestinal discomfort potentially caused by excessive α-amylase inhibition.

#### 2.3.3. Establishment of IR-HepG2 Cell Model

**Figure 10:**
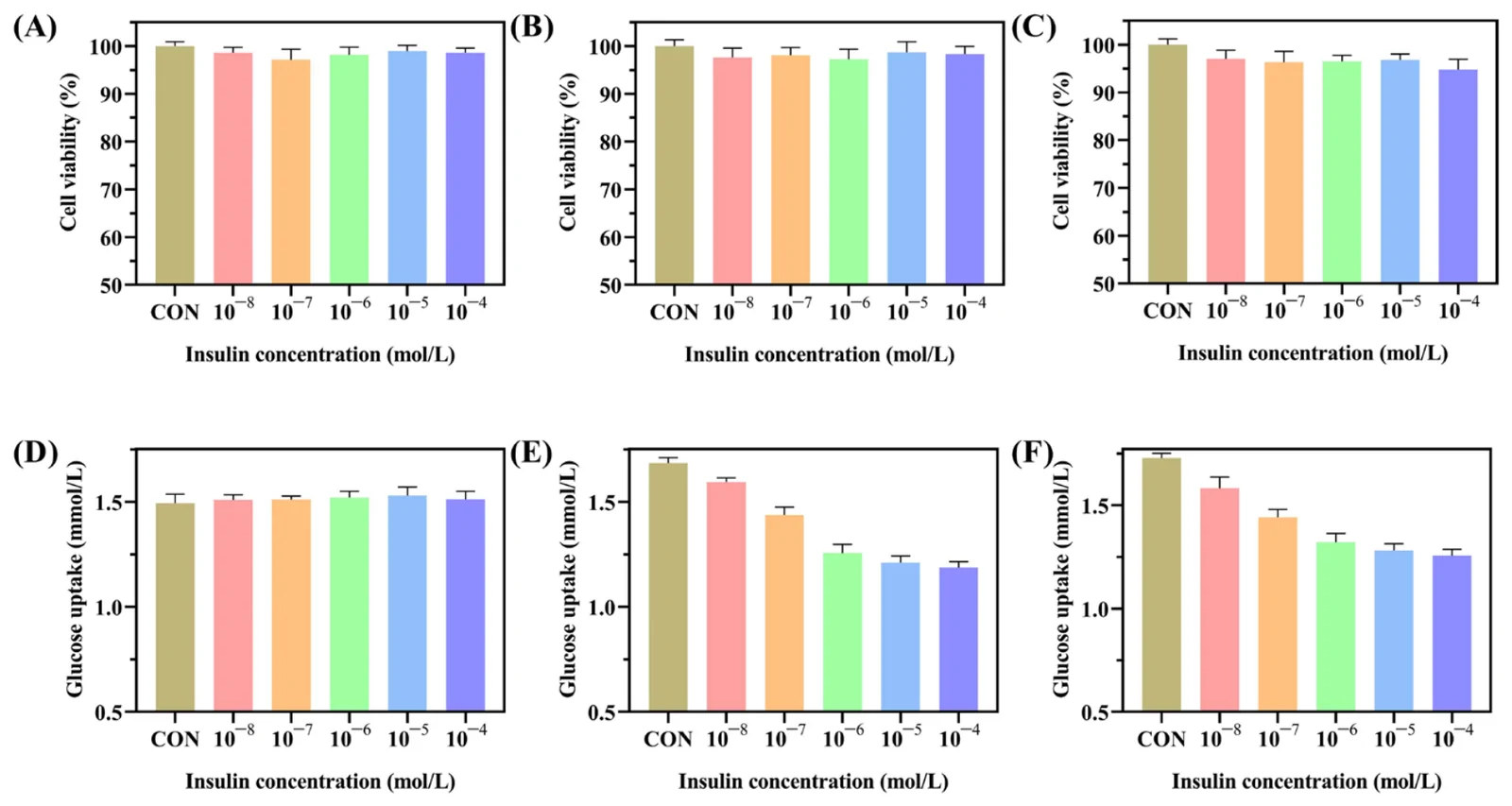
Screening of IR-HepG2 modeling conditions. (**A**–**C**) Cell viability after incubation with different concentrations of insulin for 12 h, 24 h, and 48 h; (**D**–**F**) Glucose uptake after incubation with different concentrations of insulin for 12 h, 24 h, and 48 h. Data are presented as mean ± SD (*n* = 6).

MTT assay results (Figure 10A–C) showed that insulin at different concentrations had little effect on HepG2 cell viability, with cell survival rates above 90% in all treatment groups, and above 97% in the 12 h group. Viability decreased only slightly with prolonged treatment, indicating no significant cytotoxicity under the tested conditions. Glucose uptake results (Figure 10D,E) revealed that short-term (12 h) insulin exposure slightly promoted glucose uptake, whereas prolonged (24 and 48 h) exposure to high insulin concentrations resulted in a marked decrease in glucose uptake, consistent with the development of insulin resistance [46]. After 24 h of treatment with 10^−6^ mol/L insulin, glucose uptake in the model group was significantly reduced compared to the control group—confirming that prolonged high-insulin exposure induced cellular desensitization to insulin. This decrease is not an inhibitory effect of insulin on normal cells, but rather a hallmark of successful establishment of the insulin-resistant phenotype. Considering both cell viability and the functional decline in glucose uptake induced by prolonged high-insulin exposure, 10^−6^ mol/L insulin for 24 h was selected as the optimal condition for establishing the IR-HepG2 cell model. Under this condition, cell viability remained high and the insulin-resistant state was stable, making it suitable for evaluating the ameliorative effect of PPTF on insulin resistance.

#### 2.3.4. Results of PPTF Concentration Screening in Cells

**Figure 11:**
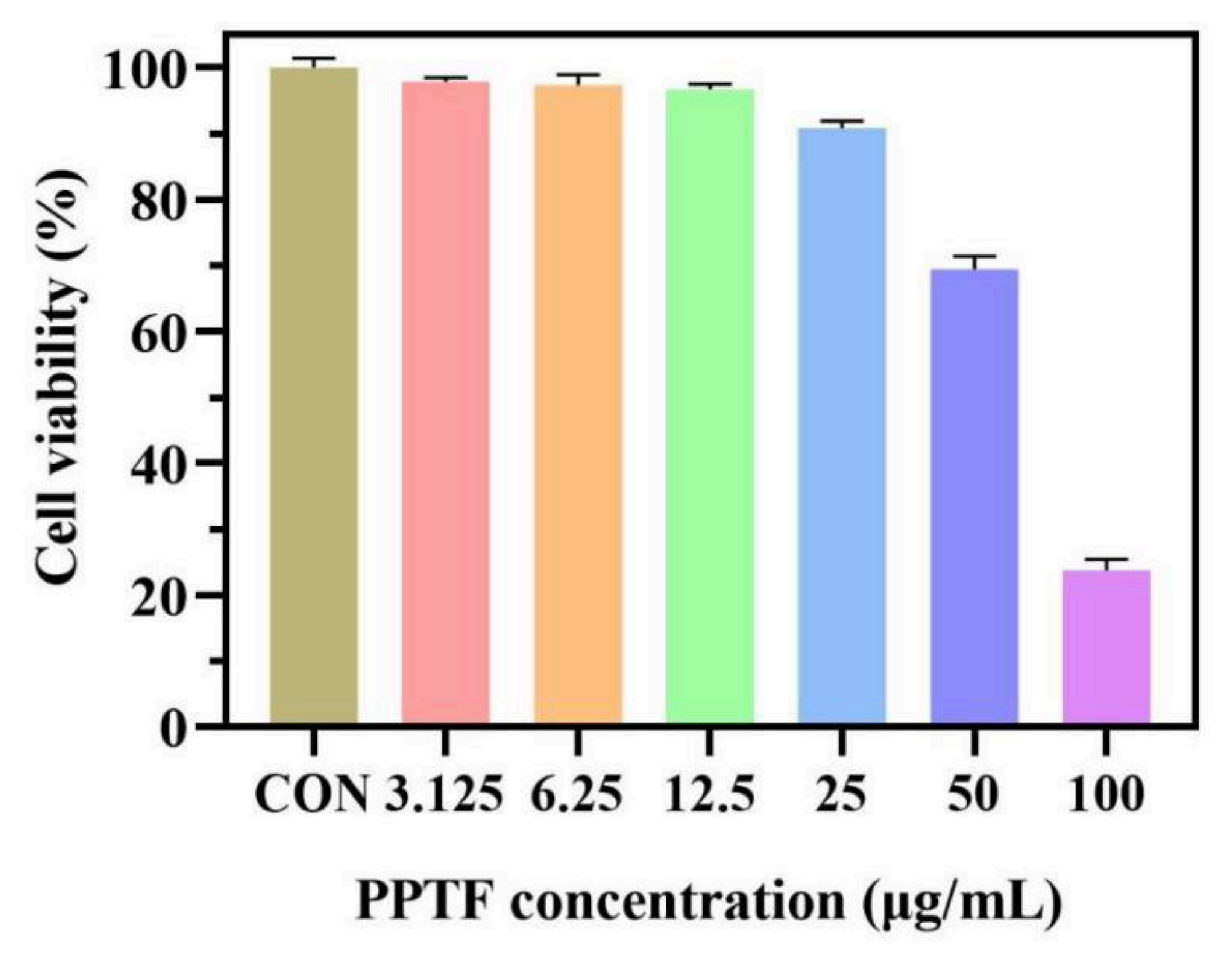
Screening results of PPTF administration concentrations. Data are presented as mean ± SD (*n* = 6).

The effect of different concentrations of PPTF on HepG2 cell viability is shown in Figure 11. PPTF demonstrated a clear dose-dependent effect on HepG2 cell viability (Figure 11). At high concentrations, significant cytotoxicity was observed, likely attributable to the pro-oxidant activity of flavonoids at supra-physiological concentrations, which can induce oxidative stress rather than antioxidant protection. In contrast, at concentrations below 12.50 μg/mL, cell viability remained above 95%, establishing a safe therapeutic window (3.13–12.50 μg/mL) for subsequent insulin resistance studies. Considering both cell viability and subsequent experimental requirements, concentrations with viability greater than 95% were selected as the safe range. Accordingly, 3.13, 6.25, and 12.50 μg/mL were determined as the low, medium, and high doses, respectively, for subsequent experiments on improving insulin resistance. This concentration range ensures good cell growth while avoiding interference from drug toxicity.

#### 2.3.5. Results of the Ameliorative Effects of PPTF on IR-HepG2 Cells

**Figure 12:**
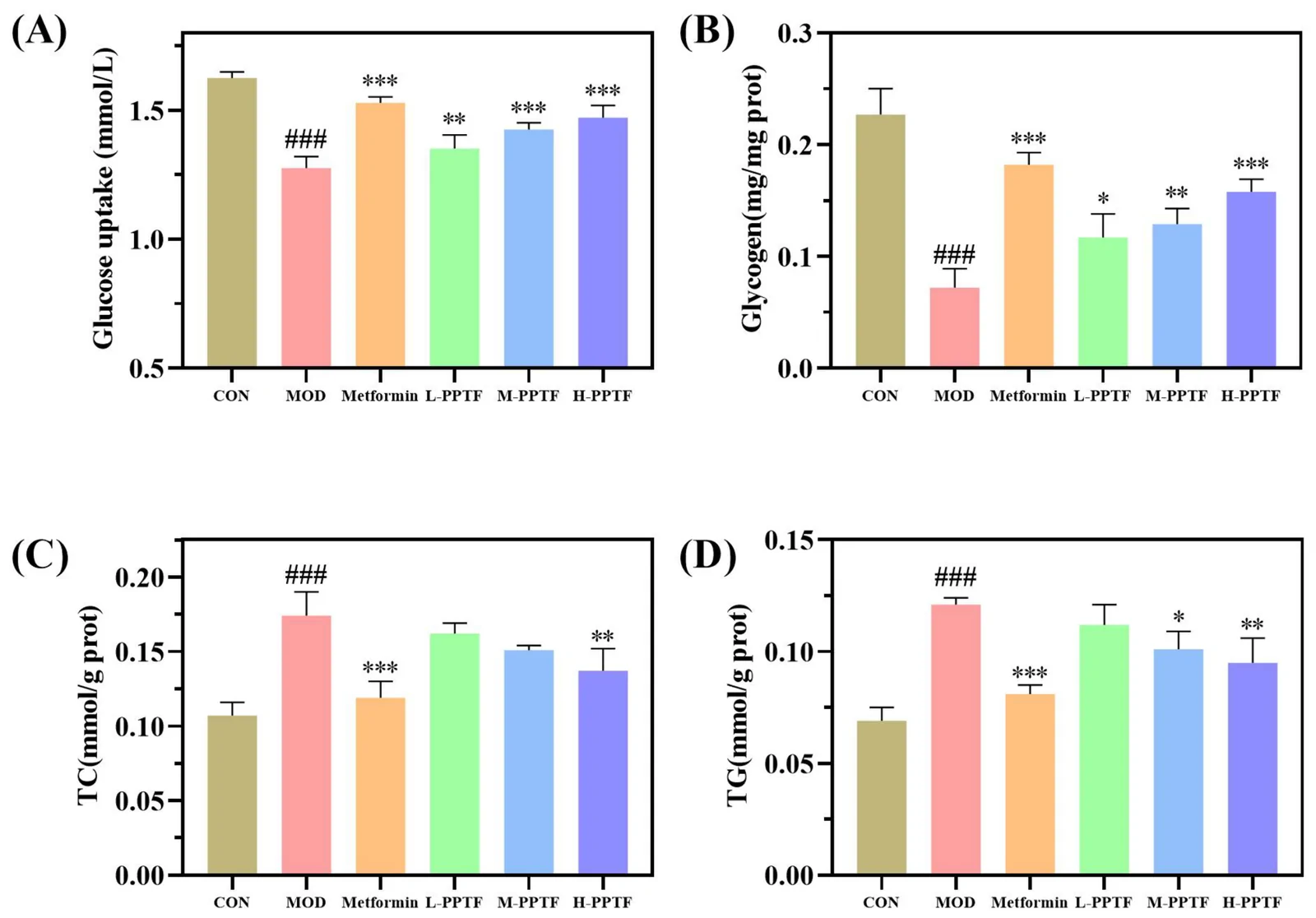
Ameliorative effects of PPTF on IR-HepG2 cells. (**A**) Ameliorative effect of PPTF on glucose uptake in IR-HepG2 cells; (**B**) Ameliorative effect of PPTF on glycogen synthesis in IR-HepG2 cells; (**C**) Ameliorative effect of PPTF on cholesterol accumulation in IR-HepG2 cells; (**D**) Ameliorative effect of PPTF on triglyceride accumulation in IR-HepG2 cells. Data are presented as mean ± SD (*n* = 6). ### *p* < 0.001 vs. CON, *** *p* < 0.001, ** *p* < 0.01 * *p* < 0.05 vs. MOD.

Glucose uptake assay (Figure 12A) showed that compared with the blank control group (1.626 mmol/L), glucose uptake in the model group significantly decreased to 1.277 mmol/L (*p* < 0.001), indicating that treatment with 10^−6^ mol/L insulin for 24 h successfully induced insulin resistance in HepG2 cells. After PPTF intervention, glucose uptake in the low-, medium-, and high-dose groups increased to 1.352, 1.425, and 1.471 mmol/L, respectively, in a dose-dependent manner, with the high-dose group showing the most significant effect. The positive control group (1.529 mmol/L) was close to that of the blank control group. Regarding glycogen synthesis (Figure 12B), glycogen content in the model group was only 31.7% of that in the blank control group. PPTF treatment restored glycogen content in the low-, medium-, and high-dose groups to 0.117, 0.129, and 0.158 mg/mg prot, respectively, representing a 119% increase in the high-dose group compared with the model group. The positive control group reached 0.182 mg/mg prot, corresponding to 80.2% of the normal level. Lipid metabolism (Figure 12C, D) was also significantly improved: Total cholesterol (TC) and triglycerides (TG) levels in the model group increased by 62.6% and 75.4%, respectively, relative to the blank control group, while PPTF treatment reduced TC and TG levels in a dose-dependent manner. In summary, PPTF dose-dependently enhanced glucose uptake and glycogen synthesis while reducing abnormal lipid accumulation in insulin-resistant HepG2 cells, indicating a beneficial effect on cellular insulin resistance at the functional level.

### 2.4. Evaluation of the In Vivo Hypoglycemic Activity of PPTF

#### 2.4.1. Ameliorative Effect of PPTF on Insulin Resistance in T2DM Mice

**Figure 13:**
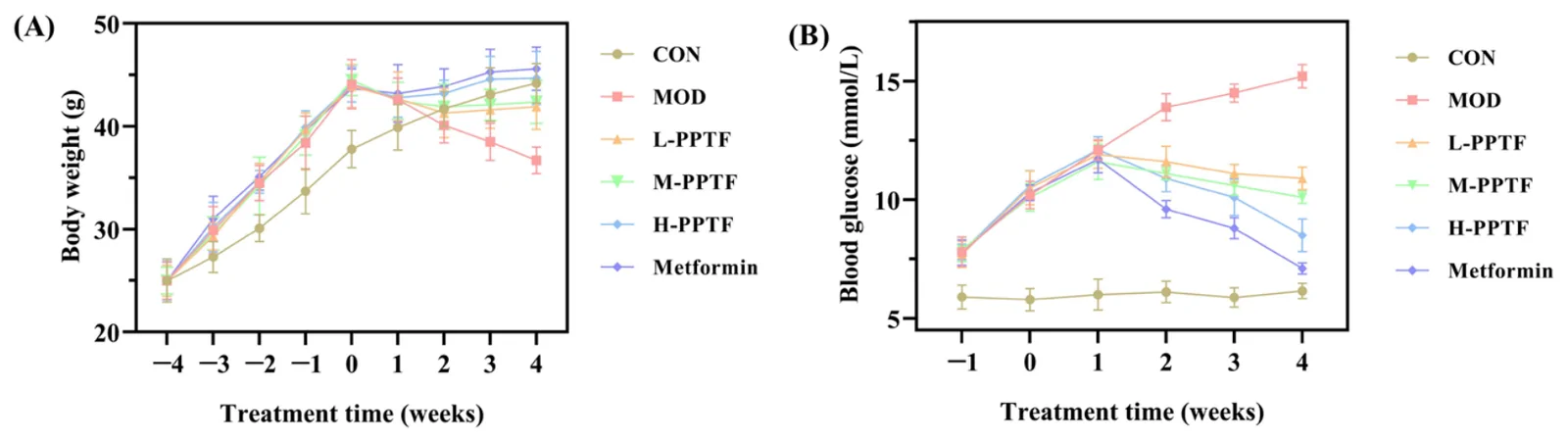
Ameliorative effects of PPTF on T2DM mice. (**A**) Amelioration of abnormal body weight loss; (**B**) Amelioration of abnormal elevation of fasting blood glucose. Data are presented as mean ± SD (blank control: *n* = 8; diabetic groups: *n* = 7).

Body weight changes are shown in Figure 13A. During the first 4 weeks before STZ injection, the body weight of the model and treatment groups increased faster than that of the blank control group due to high-fat diet feeding [47]. After STZ injection, the blank control group continued to gain weight steadily, whereas the model group showed a gradual decrease, a hallmark of the catabolic state characteristic of uncontrolled diabetes [48]. PPTF treatment alleviated diabetes-induced weight loss in a dose-dependent manner. Fasting blood glucose changes (Figure 13B) showed that after STZ injection, blood glucose levels in the model and treatment groups increased sharply, confirming successful model establishment [49]. In the following weeks, blood glucose in the model group continuously increased, whereas PPTF treatment dose-dependently reduced fasting blood glucose levels, with the high-dose PPTF group showing a significant reduction compared to the model group (*p* < 0.01). These effects suggest that PPTF provides glycemic control and may protect against the catabolic state associated with diabetes, potentially through improved insulin sensitivity and preservation of pancreatic β-cell function.

**Figure 14:**
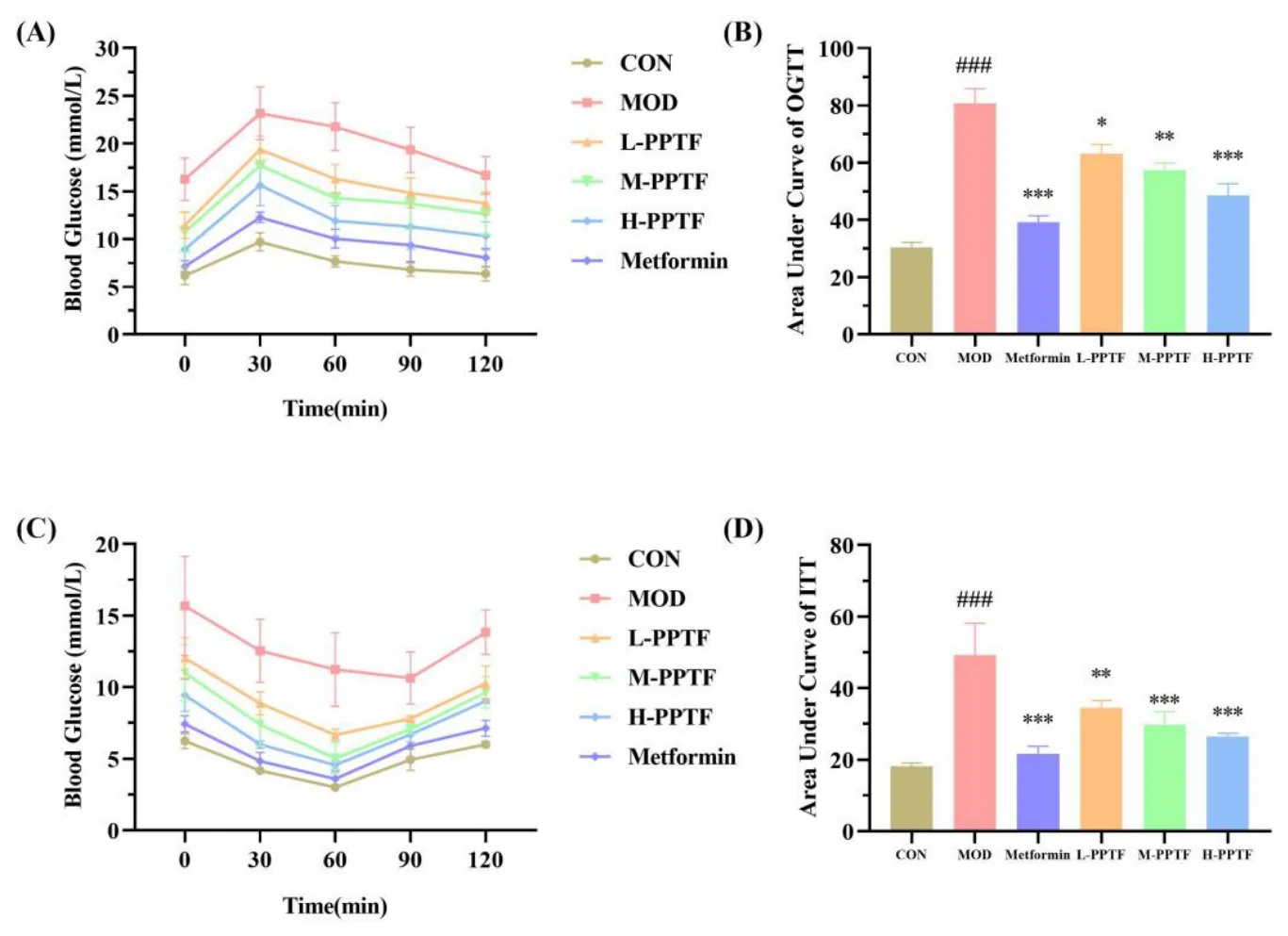
Ameliorative effects of PPTF on glucose tolerance and insulin resistance in T2DM mice. (**A**) Oral glucose tolerance test results in mice; (**B**) Area under the curve of oral glucose tolerance test in mice; (**C**) Ameliorative effect of PPTF on insulin resistance in mice; (**D**) Area under the curve of insulin resistance improvement in mice. Data are presented as mean ± SD (blank control: *n* = 8; diabetic groups: *n* = 7) ### *p* < 0.001 vs. CON, *** *p* < 0.001, ** *p* < 0.01 * *p* < 0.05 vs. MOD.

The oral glucose tolerance test (OGTT, Figure 14A) showed that in the blank control group, blood glucose peaked at 9.7 mmol/L 30 min after glucose administration and returned to baseline at 120 min. In contrast, the model group exhibited significantly elevated blood glucose, with a peak of 23.1 mmol/L at 30 min, and levels remained much higher than normal at 120 min, indicating severe glucose intolerance. PPTF treatment at all doses reduced both the peak glucose levels and the area under the curve in a dose-dependent manner. In the high-dose PPTF group, blood glucose was 15.6 mmol/L at 30 min and decreased to 10.3 mmol/L at 120 min. The positive control group showed a 120 min glucose level close to that of the blank control group. The insulin tolerance test (ITT, Figure 14C) revealed that in the blank control group, blood glucose rapidly decreased to 4.16 mmol/L at 30 min and further to 3 mmol/L at 60 min after insulin injection. The model group showed only a modest decrease from 15.6 mmol/L to 12.5 mmol/L at 30 min, indicating severe insulin resistance. In the high-dose PPTF group, blood glucose dropped to 6 mmol/L at 30 min and further to 4.5 mmol/L at 60 min, an effect comparable to that of the positive control group. These results demonstrate that PPTF effectively ameliorates impaired glucose tolerance and enhances peripheral insulin sensitivity in diabetic mice.

#### 2.4.2. Results of Serum Lipid and Lipoprotein Levels in Mice

**Figure 15:**
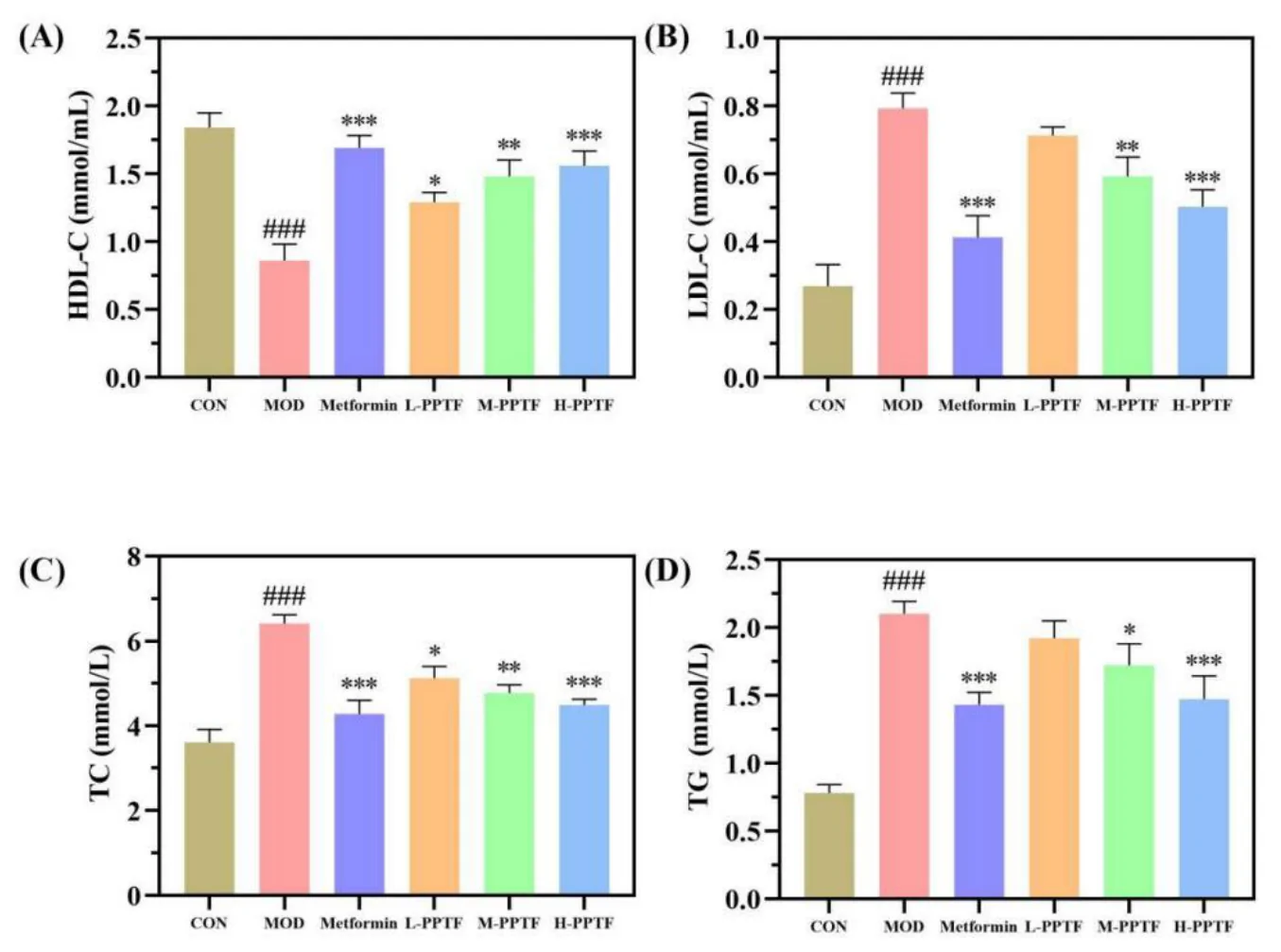
Results of serum lipid and lipoprotein determination. (**A**) PPTF improves HDL-C content in mouse serum; (**B**) PPTF improves LDL-C content in mouse serum; (**C**) PPTF improves total cholesterol content in mouse serum; (**D**) PPTF improves triglyceride content in mouse serum. Data are presented as mean ± SD (blank control: *n* = 8; diabetic groups: *n* = 7). ### *p* < 0.001 vs. CON, *** *p* < 0.001, ** *p* < 0.01 * *p* < 0.05 vs. MOD.

As shown in Figure 15, PPTF dose-dependently reversed the dyslipidemia observed in diabetic mice, as evidenced by increased high-density lipoprotein cholesterol (HDL-C) and decreased low-density lipoprotein cholesterol (LDL-C), TC, and TG levels. The correction of this lipid triad is clinically significant because diabetic dyslipidemia, characterized by low HDL-C and elevated TC/TG, is a major driver of atherosclerosis. The high-dose PPTF group showed effects comparable to the positive control metformin. By restoring lipid homeostasis, PPTF may confer cardiovascular protective benefits beyond glycemic control alone.

#### 2.4.3. Results of PPTF on Improving Glucose Metabolism in T2DM Mice

**Figure 16:**
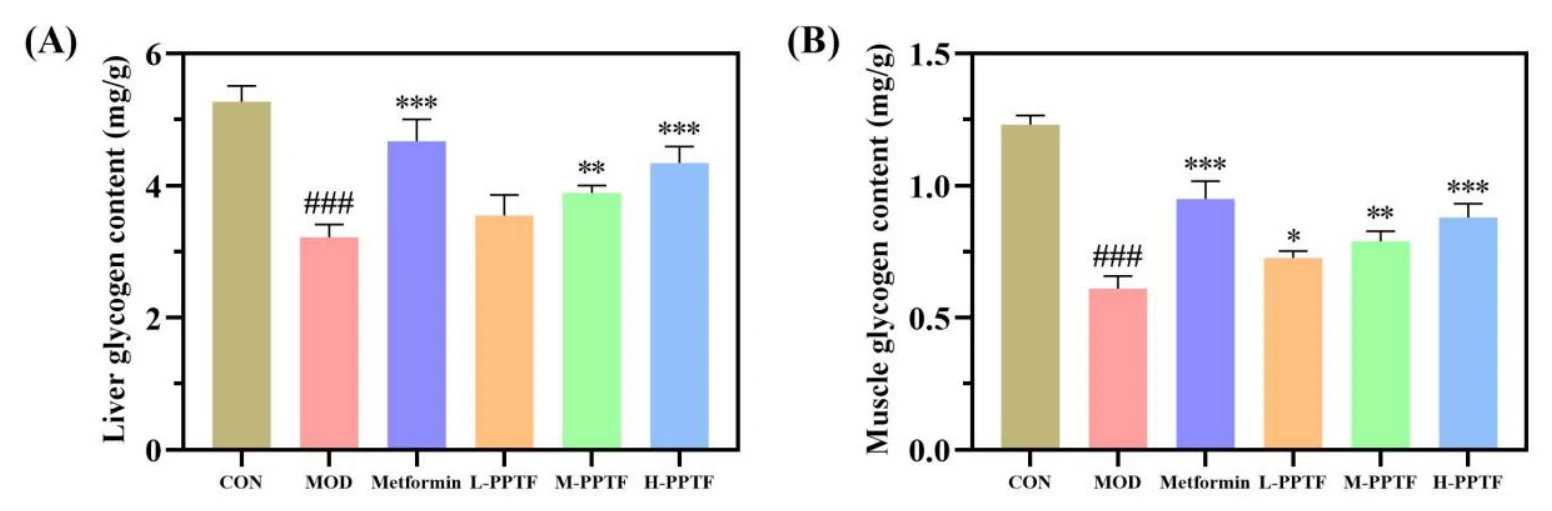
Ameliorative effect of PPTF on glycogen synthesis function in mice. (**A**) Hepatic glycogen; (**B**) Muscle glycogen. Data are presented as mean ± SD (*n* = 4). ### *p* < 0.001 vs. CON, *** *p* < 0.001, ** *p* < 0.01 * *p* < 0.05 vs. MOD.

The results of PPTF on improving hepatic and muscle glycogen metabolism in T2DM mice are shown in Figure 16. PPTF increased the reserves of hepatic glycogen and muscle glycogen in T2DM mice in a dose-dependent manner. Compared with the blank control group, hepatic glycogen (3.22 mg/g) and muscle glycogen (0.61 mg/g) in the model group were significantly reduced, indicating impaired glycogen synthesis. After PPTF intervention, the high-dose group increased hepatic glycogen to 4.34 mg/g and muscle glycogen to 0.88 mg/g, approaching the levels of the positive control metformin (4.67 mg/g and 0.95 mg/g, respectively), suggesting that PPTF promotes glycogen synthesis and ameliorates glucose metabolism disorders.

#### 2.4.4. Effects of PPTF on Histopathological Changes in T2DM Mice

**Figure 17:**
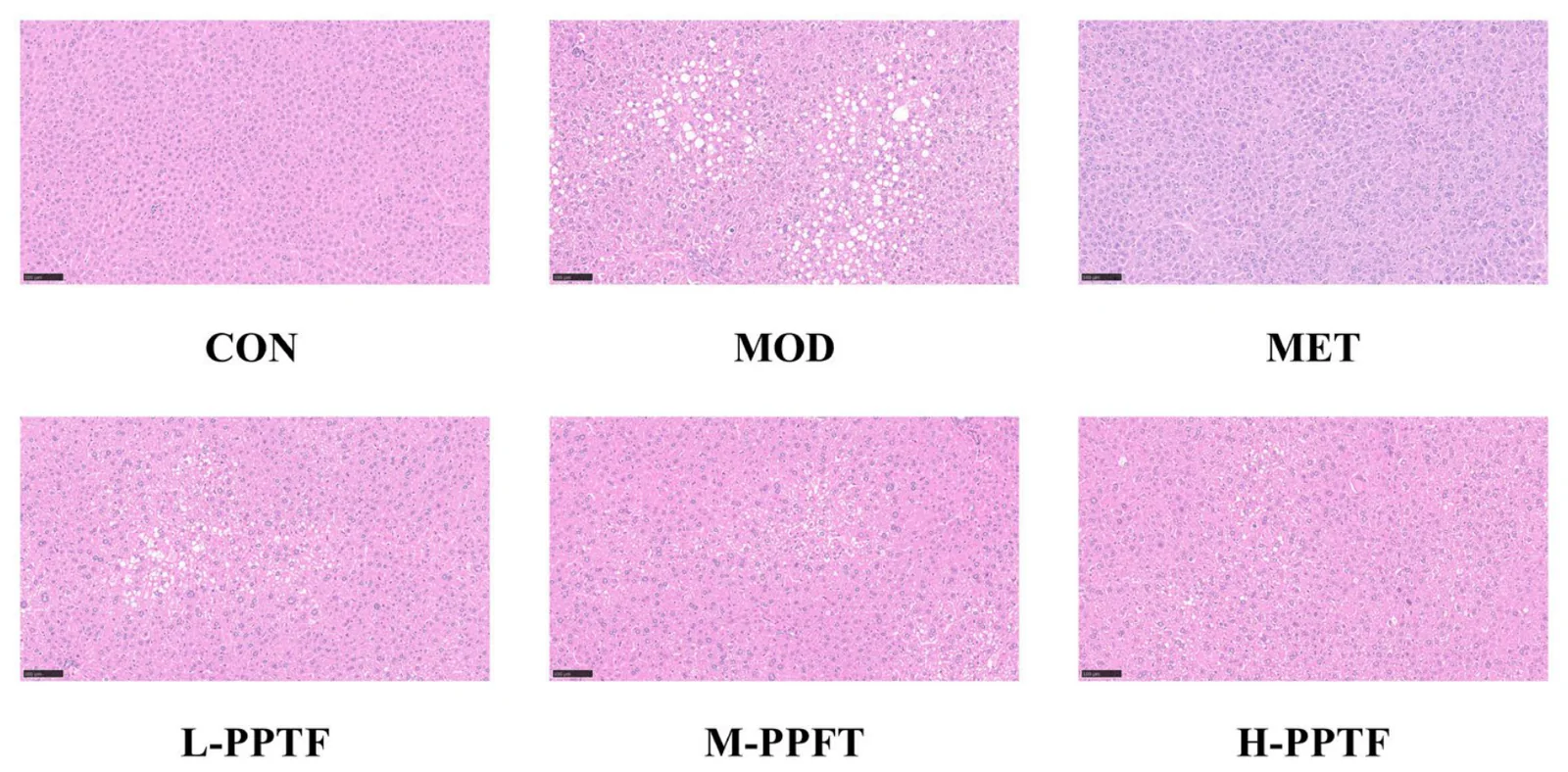
Ameliorative effect of PPTF on liver injury in mice.

**Figure 18:**
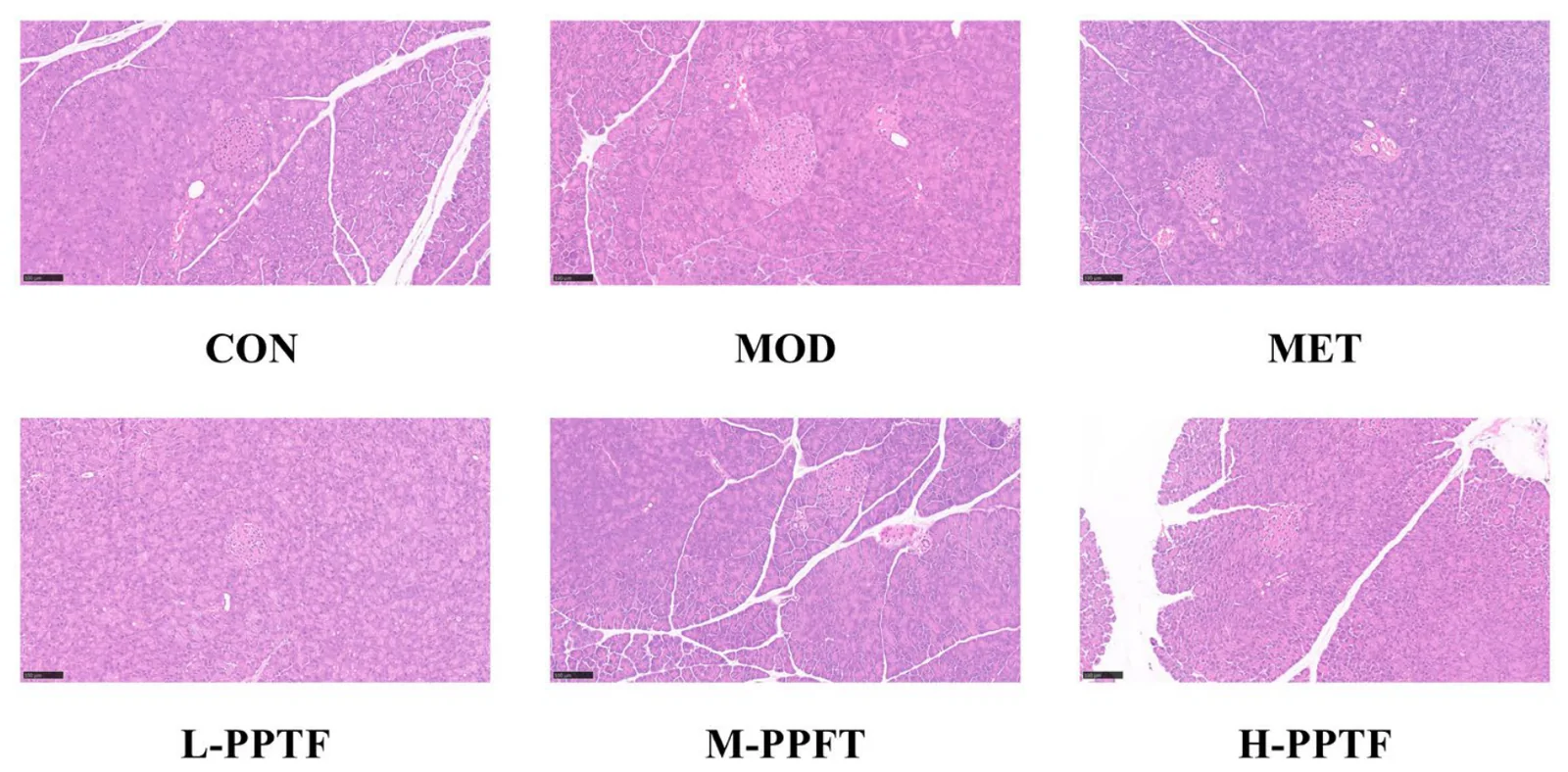
Ameliorative effect of PPTF on pancreatic islet cell injury in mice.

Liver histopathology (Figure 17) showed that in the normal control mice, hepatocytes were arranged neatly with no visible lipid vacuoles. In the model group, numerous lipid vacuoles of varying sizes appeared in hepatocytes, indicating marked steatosis [50]. After PPTF intervention, lipid vacuoles were significantly reduced, hepatocyte arrangement became more orderly, and the morphology gradually recovered, although a few vacuoles remained. Pancreatic histology (Figure 18) revealed that in normal mice, the islets were round or oval with clear boundaries and tightly packed cells. In the model group, islet boundaries were blurred, the islet cells were loosely arranged with disorganized structure and vacuoles, and there was compensatory islet enlargement [51]. Following PPTF treatment, the islet structure became more intact, the number of islet cells increased, and compensatory hypertrophy was alleviated, suggesting that PPTF may delay the progressive decline of islet function by reducing the metabolic burden on the islets.

#### 2.4.5. Summary of In Vitro and In Vivo Findings

Collectively, the in vitro and in vivo results demonstrate that PPTF exhibits multiple pharmacological effects relevant to glucose homeostasis: it shows antioxidant activity and selective α-glucosidase inhibition in vitro; it improves glucose uptake and glycogen synthesis in insulin-resistant cells; and it significantly lowers fasting blood glucose, improves glucose tolerance and insulin sensitivity, regulates serum lipids, and alleviates histopathological damage in T2DM mice. These dose-dependent effects are consistent across in vitro and in vivo experiments, suggesting that PPTF has potential as an adjuvant functional product for glycemic management.

### 2.5. Discussion

The optimized extraction yielded 39.51 mg/g of total flavonoids, with purity increasing from 33.9% to 71.2% after HPD-100 purification. These values compare favorably with previous reports on *P. perfoliatum*, where the yield was 14.98 mg/g and purity reached 43.0% [11]. The superior performance likely reflects the synergistic effects of ionic liquid-assisted ultrasound extraction and optimized gradient elution.

The hypoglycemic effects of PPTF in HFD/STZ-induced diabetic mice are consistent with findings for other flavonoid interventions, such as those from *Lycium barbarum* fruits [52]. The dose-dependent reduction in fasting blood glucose (from 15.2 to 8.5 mmol/L at 200 mg/kg) and improvements in glucose tolerance and insulin sensitivity align with response patterns reported for other flavonoid-rich extracts. Notably, PPTF at the high dose was comparable to metformin, supporting its potential as an adjunctive candidate for glycemic management.

Although PPTF exhibited cytotoxicity to HepG2 cells at 50.00–100.00 μg/mL in vitro, the in vivo dose reached 200 mg/kg/day. This apparent discrepancy can be explained by the low oral bioavailability of flavonoids due to extensive first-pass metabolism and limited intestinal absorption. The effective concentration at target tissues in vivo likely falls within the non-cytotoxic range observed in vitro.

Previous studies have shown that *P. perfoliatum* exerts hepatoprotective effects through PPARs/CPT1A/CPT2-mediated fatty acid β-oxidation [47]. The present findings extend this understanding by demonstrating hypoglycemic effects accompanied by antioxidant activity, selective α-glucosidase inhibition, and functional improvement of insulin resistance. However, the precise molecular mechanisms—particularly IRS-1/PI3K/Akt, AMPK, and GLUT4 signaling—remain to be elucidated. Limitations include incomplete chemical characterization and the exclusive use of HepG2 cells. Nonetheless, these findings provide a foundation for further development of PPTF as a candidate adjuvant functional product.

## 3. Materials and Methods

### 3.1. Materials

1-Ethyl-3-methylimidazolium bromide ([Emim]Br), 1-butyl-3-methylimidazolium bromide ([C_4_mim]Br), 1-hexyl-3-methylimidazolium bromide ([C_6_mim]Br), 1-octyl-3-methylimidazolium bromide ([C_8_mim]Br), and ABTS were purchased from Shanghai Aladdin Biochemical Technology Co., Ltd. (Shanghai, China).Rutin, hyperoside, kaempferol-3-O-rutinoside, and quercetin reference standards were purchased from Shanghai Shifeng Technology Co., Ltd. (Shanghai, China).PD-100 macroporous adsorption resin was purchased from Anhui Sanxing Resin Technology Co., Ltd. (Bengbu, China). PH, α-glucosidase, DMEM high-glucose medium, recombinant human insulin, p-NPG, and the BCA protein assay kit were purchased from Beijing Solarbio Science & Technology Co., Ltd. (Beijing, China). Total cholesterol, triglyceride, muscle/liver glycogen, low-density lipoprotein cholesterol, high-density lipoprotein cholesterol, and glucose assay kits were purchased from Nanjing Jiancheng Bioengineering Institute. The human hepatocellular carcinoma (HepG2) cell line was purchased from the Shanghai Institute of Cell Biology, Chinese Academy of Sciences (Shanghai, China). TPTZ was purchased from Shanghai Macklin Biochemical Technology Co., Ltd. (Shanghai, China). Streptozotocin was purchased from Tianjin Yongda Chemical Reagent Co., Ltd. (Tianjin, China). α-Amylase and soluble starch were purchased from Tianjin Zhonglian Chemical Reagent Co., Ltd. (Tianjin, China). Male Kunming mice (6 weeks old, 20–22 g) were purchased from Shanxi Medical University, license number: SYXK (Jin) 2024-0007. All other reagents and solvents were of analytical grade.

### 3.2. Methods

#### 3.2.1. Experimental Method for PPTF Extraction

##### Preparation of the Rutin Standard Curve

The total flavonoid content was determined by the aluminum trichloride colorimetric method. A rutin standard solution (0.156 g/L) was prepared in 80% ethanol. Aliquots of the standard solution (0.4, 0.6, 0.8, 1.0, 1.2, 1.4, 1.6, 1.8, 2.0, 2.2, and 2.4 mL) were transferred into 10 mL volumetric flasks. Color development was initiated by adding 4 mL of 1 mol/L sodium acetate and 3 mL of 0.1 mol/L aluminum trichloride. Each mixture was brought to volume with 80% ethanol, thoroughly mixed, and the absorbance was recorded at 412 nm. A calibration curve was constructed by plotting absorbance (Y) against rutin concentration (X).

##### Determination of PPTF Content

For PPTF content determination, 0.25 mL of the test solution was combined with 4 mL of 1 mol/L sodium acetate and 3 mL of 0.1 mol/L aluminum trichloride in a 10 mL volumetric flask. The mixture was adjusted to volume with 80% ethanol, homogenized, and the absorbance was measured at 412 nm. The total flavonoid content was calculated using the following formula:

(1) Total flavonoid content (mg/g) = (C × N × V)/m

where C is the concentration of PPTF (mg/mL), N is the dilution factor, V is the volume of the extract (mL), and m is the mass of *Polygonum perfoliatum* powder (g).

##### Single-Factor Experiments for PPTF Extraction

The dried aerial parts of *P. perfoliatum* were pulverized and passed through an 80-mesh sieve. For ionic liquid screening, each of the four tested ionic liquids was dissolved in 60% ethanol at a concentration of 8 g/L. Extractions were performed using 1.00 g of plant powder under fixed conditions: solid-to-liquid ratio 1:30 (g/mL), temperature 60 °C, ultrasonic power 225 W, and duration 50 min. After identifying the optimal ionic liquid, the effects of six parameters on PPTF yield were individually evaluated: ethanol concentration (50–90%), ionic liquid concentration (2–10 g/L), ultrasonic power (75–375 W, equivalent to 5–25% of the 1500 W maximum), extraction time (30–70 min), solid-to-liquid ratio (1:10–1:30), and extraction temperature (30–70 °C).

##### Optimization of PPTF Extraction Conditions by Response Surface Methodology

Based on the single-factor experimental results, three independent variables that exhibited significant effects on the extraction yield were selected for further optimization. A three-factor, three-level Box–Behnken design (Table 5) was employed with the extraction yield as the response variable. A total of 17 experimental runs were conducted to optimize the ultrasound-assisted ionic liquid extraction of total flavonoids. The experimental data were analyzed using Design-Expert software (version 13, Stat-Ease, Inc., Minneapolis, MN, USA), and the optimal extraction conditions were subsequently validated.

#### 3.2.2. Purification of PPTF by Macroporous Resin

##### Resin Pretreatment

Prior to use, the macroporous adsorption resin was pretreated to remove residual monomers, porogens, and other impurities that could compromise adsorption performance. The resin was soaked in 95% ethanol for 24 h to facilitate swelling and dissolution of organic residues, followed by repeated washing with distilled water until the ethanol odor was no longer detectable. For wet-packing, the ethanol-conditioned resin was packed into the column and eluted with 95% ethanol until the effluent remained clear upon mixing with water, ensuring complete removal of lipophilic contaminants. The column was subsequently rinsed with purified water until ethanol-free, completing the solvent exchange from organic to aqueous phase.

##### Plotting of the Dynamic Adsorption Breakthrough Curve

Dynamic adsorption experiments were conducted using a glass column (φ = 20 mm, diameter-to-height ratio 1:10) packed with 20 mL of pretreated HPD-100 resin. The flavonoid extract (2.0 mg/mL) was loaded at flow rates of 1, 2, and 3 BV/h. Effluent fractions of 0.5 BV were collected and analyzed for flavonoid content. The breakthrough point was defined as the effluent concentration reaching 10% of the feed concentration; the cumulative volume loaded up to this point was taken as the maximum effective loading volume at each flow rate. The optimal flow rate and loading capacity were determined by comparing the breakthrough profiles across the three flow rates.

##### Gradient Elution Method

After sample loading, the column was sequentially eluted with 2.5 BV each of distilled water, 10%, 30%, 60%, 80%, and 95% ethanol at a flow rate of 2 BV/h. The eluate was collected in 0.25 BV fractions, and flavonoid concentrations were determined to construct a gradient elution profile. The elution peaks at varying ethanol concentrations were compared to identify the primary elution zone for the target flavonoids, which served as a guide for subsequent isocratic elution optimization.

##### HPLC Analysis Method for Flavonoid Identification and Quantification

An HPLC method was established for the identification and quantification of major flavonoids in both the crude extract and the purified lyophilized powder of *P. perfoliatum*, and was validated for stability, repeatability, and recovery. Chromatographic separation was performed on an Agilent ZORBAX SB C18 column (4.6 × 150 mm, 5 µm) at 30 °C, with a mobile phase of methanol (A) and 0.4% phosphoric acid (B) under gradient elution (0–32 min, 40%→52% A; 32–48 min, 52%→55% A; 48–60 min, 55%→65% A) at 1.0 mL/min. The detection wavelength was 360 nm and the injection volume was 10 µL. A mixed standard solution was prepared by dissolving hyperoside (6.812 mg), kaempferol-3-O-rutinoside (3.312 mg), and quercetin (5.025 mg) in 70% methanol to a final volume of 10 mL, giving concentrations of 681.20, 331.20, and 502.50 μg/mL, respectively. For sample preparation, 1 g of each lyophilized powder (pre- and post-purification) was accurately weighed, dispersed in 10 mL of 70% methanol, ultrasonicated for 30 min, and reweighed to adjust the volume with 70% methanol. The mixture was centrifuged at 2000 r/min for 5 min, and the supernatant was filtered through a 0.45 μm membrane before injection.

#### 3.2.3. In Vitro Activity Evaluation of PPTF

##### Methods for the Evaluation of In Vitro Antioxidant Activity

PPTF was dissolved in deionized water to obtain a series of solutions at concentrations of 1000.00, 500.00, 250.00, 125.00, 62.50, 31.25, 15.63, and 7.81 μg/mL. A 0.8 mg/mL DPPH solution was prepared in anhydrous ethanol. The reaction mixture, consisting of 2 mL of DPPH solution and 2 mL of each PPTF dilution, was incubated in the dark at room temperature for 30 min, and the absorbance was recorded at 517 nm. Vitamin C at equivalent concentrations was used as the positive control. Deionized water replaced the PPTF solution in the control group and the DPPH solution in the blank group.

The ABTS^+^ working solution was prepared by dissolving 0.0384 g of ABTS in 10 mL of PBS (pH 7.4) and 0.0134 g of potassium persulfate (K_2_S_2_O_8_) in 10 mL of deionized water. The two solutions were mixed in a 1:1 (*v*/*v*) ratio and stored in the dark at 4 °C for more than 16 h to allow complete generation of ABTS^+^. Prior to use, the resulting solution was diluted with PBS to an absorbance of 0.2–0.8 at 734 nm. PPTF was serially diluted to concentrations of 125.00, 62.50, 31.25, 15.63, 7.81, and 3.91 μg/mL. In a 96-well plate, 40 μL of each PPTF dilution was mixed with 160 μL of ABTS^+^ working solution as the sample group (A_s_). The control group (A_C_) replaced the PPTF solution with deionized water, and the blank group (A_0_) replaced ABTS^+^ with deionized water. After incubation at room temperature for 6 min, the absorbance was measured at 734 nm. The scavenging rate for both DPPH and ABTS assays was calculated using the following formula:

(2) $$\mathrm{Scavenging}\;\mathrm{rate}\left(\%\right)\;=\;(1-\frac{A_{s}-A_{0}}{A_{c}})\;\times \;100\%$$

where A_S_ is sample absorbance, A_C_ is control absorbance, and A_0_ is blank absorbance.

The FRAP reagent was prepared by combining 300 mmol/L acetate buffer (0.1821 g sodium acetate and 1.585 mL glacial acetic acid, diluted to volume), 20 mmol/L FeCl_3_·6H_2_O, and 10 mmol/L TPTZ solution (0.1562 g TPTZ and 0.085 mL concentrated HCl, diluted to 50 mL) in a 10:1:1 (*v*/*v*/*v*) ratio. PPTF was serially diluted to concentrations of 500.00, 250.00, 125.00, 62.50, 31.25, and 15.63 μg/mL. An aliquot of 0.2 mL of each PPTF solution was mixed with 3.9 mL of the FRAP reagent and incubated in a water bath at 37 °C for 10 min. The absorbance was then measured at 593 nm. Vitamin C solutions at the same concentrations served as the positive control. A standard curve was constructed using FeSO_4_·7H_2_O at concentrations ranging from 0.125 to 2 mmol/L. The total antioxidant capacity of the samples was expressed as FeSO_4_ equivalents (mmol/L).

##### PPTF Enzyme Inhibition Assay Methods

For α-glucosidase inhibition, PPTF was serially diluted to concentrations of 125.00, 62.50, 31.25, 15.63, 7.81, and 3.91 μg/mL, with acarbose as the positive control. In a 96-well plate, 40 μL of each PPTF dilution was mixed with 40 μL of α-glucosidase (1 U/L) and incubated at 37 °C for 10 min. Subsequently, 40 μL of p-nitrophenyl-α-D-glucopyranoside (PNPG) substrate was added, and the reaction was allowed to proceed for 20 min before being terminated by the addition of 80 μL of 0.2 mol/L Na_2_CO_3_. Absorbance was measured at 400 nm. A sample control (enzyme replaced by PBS), a blank (sample replaced with distilled water), and a blank control (both sample and enzyme replaced with PBS) were included.” The inhibition rate was calculated as:

(3) $$\mathrm{Inhibition}\;(\%)\;=\;1-\frac{A_{s}-A_{c}}{A_{1}-A_{0}}\;\times \;100\%$$

where A_s_ is sample absorbance, A_c_ is sample control, A_1_ is blank, and A_0_ is blank control.

PPTF was diluted to 1000.00, 500.00, 250.00, 125.00, and 62.50 μg/mL. Acarbose was used as a positive control. A 0.125 mL aliquot of α-amylase (1 U/L) was mixed with an equal volume of PPTF solution and incubated at 37 °C for 30 min. Then, 0.25 mL of 1% starch solution was added and the mixture was incubated at 37 °C for another 5 min. DNS color reagent was added, followed by heating in a boiling water bath for 10 min. The mixture was cooled and diluted to 10 mL with distilled water, and the absorbance was measured at 520 nm. The control group replaced the PPTF solution with PBS. The inhibition rate was calculated as:

(4) $$\mathrm{Inhibition}\left(\%\right)\;=\;\frac{A_{0}-A_{s}}{A_{0}}\;\times \;100\%$$

where A_s_ is sample absorbance and A_0_ is control absorbance.

##### Experimental Method for the Ameliorative Effect of PPTF on IR-HepG2 Cells

HepG2 cells were cultured in DMEM high-glucose medium supplemented with 10% fetal bovine serum (FBS) and 1% penicillin-streptomycin at 37 °C in a 5% CO_2_ incubator. Cells were passaged upon reaching approximately 90% confluence by trypsinization and resuspension. For cryopreservation, cells were stored in DMEM containing 15% FBS and 10% DMSO, frozen in a controlled-rate cooling container at −80 °C, and subsequently transferred to liquid nitrogen for long-term storage. For cell cycle synchronization, cells seeded in 96-well plates were serum-starved in DMEM without FBS for 12 h. Insulin resistance was induced by exposing the cells to graded concentrations of insulin (10^−8^–10^−4^ mol/L) for 12, 24, or 48 h.

Cell viability was assessed using the MTT assay. After treatment, 10 μL of MTT solution (5 mg/mL) was added to each well and the plates were incubated for 4 h. The resulting formazan crystals were dissolved in 100 μL of DMSO, and the absorbance was read at 570 nm. Cell viability was calculated using the following formula:

(5) $$\mathrm{Viability}\;(\%)\;=\;\frac{A_{s}-A_{0}}{A_{c}-A_{0}}\;\times \;100\%$$

where A_s_ is the absorbance of test well, A_c_ is control well (untreated cells), and A_0_ is blank well (medium only).

Glucose levels in the culture medium were measured using a commercial glucose assay kit. Following the addition of the reagents, the plate was incubated at 37 °C for 10 min, and the absorbance was recorded at 505 nm. The glucose concentration was calculated according to the formula provided by the kit manufacturer.

(6) $$\mathrm{Glucose}\;\mathrm{concentration}\;\left(\text{mmol/L}\right)=\frac{A_{s}-A_{0}}{A_{\mathrm{std}}-A_{0}}\;\times {\;C}_{\mathrm{std}}\;÷\;\mathrm{Cpr}$$

where A_s_ is the absorbance of the sample, A_std_ is the absorbance of the standard solution, A_0_ is the absorbance of the blank well, C_std_ is the standard concentration (5.55 mmol/L), and Cpr is the protein concentration (gprot/L).

To evaluate the cytotoxicity of PPTF, HepG2 cells were exposed to graded concentrations (3.13, 6.25, 12.50, 25.00, 50.00, and 100.00 μg/mL) for 24 h, and cell viability was determined by the MTT assay. Concentrations that maintained cell viability above 95% (i.e., 3.13, 6.25, and 12.50 μg/mL) were considered non-cytotoxic and were therefore selected for subsequent experiments.

For the assessment of glucose consumption, six experimental groups were established (*n* = 6 per group): a blank control group (serum-free DMEM), a model group (10^−6^ mol/L insulin), three PPTF treatment groups (insulin plus 3.13, 6.25, or 12.50 μg/mL PPTF), and a positive control group (insulin plus 2 mg/L metformin). Cells were first serum-starved in DMEM without FBS for 24 h, and then incubated with the respective treatments for an additional 24 h. Glucose consumption in the culture medium was subsequently measured. In parallel, cells cultured in 6-well plates were subjected to the same treatment regimen, and after 24 h they were lysed for further biochemical analyses.

For glycogen determination, the collected cell pellets were lysed with an alkaline solution, heated in a boiling water bath for 20 min, and diluted to obtain a glycogen test solution. Glycogen content was measured using a commercial kit at 620 nm and was normalized to the protein concentration determined by the BCA method. Intracellular total cholesterol (TC) and triglyceride (TG) levels were similarly measured using commercial kits, with absorbance read at 500 nm, and the results were also normalized to protein content.

(7) $$\mathrm{Total}\;\mathrm{glycogen}\;\mathrm{content}\;\left(\text{mg/mgprot}\right)=\frac{A_{s}}{A_{\mathrm{std}}}\;\times \;C_{\mathrm{std}}\;\times \;10\;÷\;1.11\;÷\;\mathrm{Cpr}$$

where A_s_ is the absorbance of the sample, A_std_ is the absorbance of the standard solution, C_std_ is the standard concentration (0.01 mg/mL), 10 is the dilution factor, 1.11 is the mass conversion coefficient between glycogen and glucose, and Cpr is the protein concentration of the cell homogenate (mgprot/mL).

(8) $$\text{TC/TG}\;\mathrm{content}\;\left(\text{mmol/gprot}\right)=\frac{A_{s}-A_{0}}{A_{\mathrm{std}}-A_{0}}\;\times {\;C}_{\mathrm{std}}\;÷\;\mathrm{Cpr}$$

where A_s_ is the absorbance of the sample, A_std_ is the absorbance of the standard solution, A_0_ is the absorbance of the blank well, C_std_ is the standard concentration (7.06 mmol/L for cholesterol and 1.31 mmol/L for triglyceride), and Cpr is the protein concentration (gprot/L).

#### 3.2.4. In Vivo Hypoglycemic Activity Evaluation of PPTF

##### Establishment of T2DM Mouse Model and Dosing Regimen

Mice were maintained in a specific pathogen-free barrier facility under controlled conditions (25 ± 2 °C, 50–70% relative humidity, 12 h light/dark cycle) with ad libitum access to food and water. After a 1-week acclimatization period, eight mice were randomly assigned to the normal control group (NC) and maintained on a standard diet, while the remaining animals were placed on a high-fat diet (HFD). Following 4 weeks of HFD feeding, the NC group received an intraperitoneal (i.p.) injection of 0.1 M citrate buffer (pH 4.4), whereas the HFD-fed mice were injected i.p. with STZ (30 mg/kg in 0.1 M sodium citrate buffer, pH 4.4) once daily for five consecutive days. One day after the first STZ injection, all mice were fasted for 6 h with free access to water, and blood samples were collected from the tail vein for fasting blood glucose (FBG) measurement. Animals with FBG ≥ 11.1 mmol/L were classified as diabetic and enrolled in subsequent experiments. A total of 35 diabetic mice were randomly assigned to five groups (*n* = 7 per group) using a randomized block design based on initial body weight: model group (vehicle, distilled water, 10 mL/kg/d), positive control group (metformin, 200 mg/kg/d), and PPTF low-, medium-, and high-dose groups (50, 100, and 200 mg/kg/d, respectively, suspended in 0.5% carboxymethyl cellulose sodium). Eight normal mice were used as the blank control group (vehicle, distilled water). The sample size was determined with reference to previous studies on similar flavonoid interventions in diabetic mouse models. Throughout the experiment, the NC group was fed a standard diet, while all other groups continued on the HFD. All treatments were administered daily by oral gavage for four consecutive weeks. To minimize potential bias, the investigator responsible for administering treatments and performing measurements was blinded to group allocation, and all data were coded before statistical analysis.

All animals were monitored daily for signs of distress or adverse events. No animals exhibited abnormal behaviors or required premature euthanasia during the study.

##### Collection and Processing of Mouse Blood and Tissue Samples

After four weeks of PPTF administration, the T2DM mice underwent OGTT and IT. Following the completion of all measurements and after the animals had recovered, deep anesthesia was induced by intraperitoneal injection of sodium pentobarbital (6 mL/kg), and the mice were subsequently sacrificed by exsanguination. Blood samples were collected via retro-orbital bleeding, allowed to clot at room temperature for 30 min, and then centrifuged at 4000 r/min for 20 min. The resulting serum was carefully separated, aliquoted, and stored at −20 °C for subsequent biochemical analyses.

For histopathological evaluation, liver and pancreas samples were collected from 4 mice per group (3 mice from the diabetic groups due to odd group size, with the allocation randomized). These tissues were rinsed with ice-cold saline, immediately fixed in 4% paraformaldehyde for 24 h at room temperature, and then routinely processed for paraffin embedding, sectioning, and hematoxylin and eosin (H&E) staining. The stained sections were mounted with neutral balsam for light microscopic observation and image acquisition. The remaining mice (4 from the blank control group and 3 from each diabetic group) were used to collect liver and gastrocnemius muscle tissues for glycogen determination. These tissues were washed with ice-cold saline and stored at −20 °C for subsequent analyses.

##### Oral Glucose Tolerance and Insulin Resistance Tests

OGTT was conducted after 4 weeks of treatment, prior to the final dose. Mice were fasted for 6 h (from 8:00 AM), and baseline blood glucose was measured (0 min). A 20% glucose solution was then administered orally at 2 g/kg, and blood glucose was monitored at 30, 60, 90, and 120 min post-administration. The glucose response curve was plotted, and the area under the curve (AUC) was calculated. After the test, food and water were resumed, and the daily treatment was administered. Three days after OGTT, the ITT was performed. Mice were fasted for 6 h, and baseline blood glucose was measured (0 min). Human recombinant insulin (0.75 U/kg) diluted in sterile saline containing 0.1% bovine serum albumin (BSA) was injected intraperitoneally. Blood glucose levels were measured at 30, 60, 90, and 120 min post-injection. Strict timing was maintained throughout. After the last blood collection, food and water were returned, and the daily drug was given.

##### Serum Lipid and Lipoprotein Detection

After collecting mouse serum using the above method, Serum levels of TC, TG, LDL-C, and HDL-C were determined using commercial assay kits according to the manufacturers’ instructions. The concentrations were calculated using the following formula:

(9) $$\mathrm{Concentration}\;\left(\text{mmol/L}\right)\;=\;\frac{A_{s}-A_{0}}{A_{\mathrm{std}}-A_{0}}\;\times {\;C}_{\mathrm{std}}$$

where A_s_ is the absorbance of the sample, A_std_ is the absorbance of the standard, A_0_ is the absorbance of the blank, and C_std_ is the standard concentration (TC: 7.06 mmol/L, TG: 1.31 mmol/L, LDL-C: 5.12 mmol/L, HDL-C: 1.54 mmol/L).

##### Effect of PPTF on Glucose Metabolism in T2DM Mice

Glycogen levels in liver and gastrocnemius muscle were measured using the anthrone colorimetric method. Tissue samples (≤100 mg) were rinsed with saline, weighed, and mixed with three volumes of alkali solution, then heated in a boiling water bath for 20 min. After cooling, 16 volumes of distilled water were added to prepare a 5% tissue homogenate. For fatty liver samples, one-quarter volume of chloroform was added for delipidation, followed by vortexing and centrifugation at 3500 r/min for 10 min; the supernatant was retained. The prepared solution was combined with the assay reagents, heated in a boiling water bath for 5 min, and the absorbance was recorded at 620 nm. Glycogen content was calculated using formula:

(10) $$\mathrm{Glycogen}\;\left(\text{mg/g}\right)\;=\;\frac{A_{s}}{A_{\mathrm{std}}}\;\times \;m_{\mathrm{std}}\;\times \;N\;÷\;1.11$$

where A_s_ is the absorbance of the sample, A_std_ is the absorbance of the standard solution, m_std_ is the standard sugar content (0.01 mg), N is the dilution factor of the sample pretreatment, and 1.11 is the conversion coefficient between glucose and glycogen in the anthrone colorimetric assay (the color intensity of 100 μg glycogen corresponds to that of 111 μg glucose).

#### 3.2.5. Statistical Analysis

All experimental results are expressed as mean ± standard deviation (SD). For cell-based assays (Section 2.3 and Section 3.2.3), data were obtained from six independent replicates (*n* = 6 per group). For animal experiments (Section 2.4 and Section 3.2.4), the sample sizes were *n* = 8 for the blank control group and *n* = 7 for each diabetic group, as specified in Section Establishment of T2DM Mouse Model and Dosing Regimen. Statistical analyses were performed using GraphPad Prism 8.0.2 software (GraphPad Software, Boston, MA, USA). Normality and homogeneity of variance were assessed using the Shapiro–Wilk test and Levene’s test, respectively; all data met the assumptions for parametric analysis. Comparisons between two groups were performed using a two-tailed Student’s *t*-test, whereas multiple group comparisons were analyzed by one-way analysis of variance (ANOVA) followed by Dunnett’s post hoc test for comparisons against the model group. A *p* value < 0.05 was considered statistically significant. The significance levels in the figures are indicated as follows: compared with the blank control group, *#*, *##*, and *###*, represent *p* < 0.05, *p* < 0.01, and *p* < 0.001, respectively; compared with the model group, *, **, and *** represent *p* < 0.05, *p* < 0.01, and *p* < 0.001, respectively.

## 4. Conclusions

In this study, the traditional Chinese medicinal herb *Polygonum perfoliatum* L. was systematically investigated, including optimization of the extraction process of total flavonoids, purification by macroporous resin, evaluation of hypoglycemic activity in vitro, and anti-diabetic efficacy in vivo. The main conclusions are as follows:

An optimal ultrasound-assisted ionic liquid extraction process for PPTF was established, achieving an extraction yield of 39.51 mg/g. After purification with HPD-100 macroporous resin, the total flavonoid content increased from 33.9% to 71.2%. The HPLC method was validated with good linearity, repeatability, stability, and recovery, confirming its suitability for the quantitative analysis of PPTF.

In vitro bioactivity evaluation demonstrated that PPTF possesses good DPPH and ABTS radical scavenging activities and FRAP total reducing power. Its inhibitory effect on α-glucosidase was stronger than that on α-amylase, showing a selective inhibition profile. PPTF showed no cytotoxicity to HepG2 cells at concentrations of 3.13–12.50 μg/mL, and improved glucose uptake, promoted glycogen synthesis, and reduced intracellular TC and TG accumulation in insulin-resistant cells in a dose-dependent manner.

In vivo pharmacodynamic studies using a high-fat diet combined with streptozotocin-induced T2DM mouse model revealed that after 4 weeks of intragastric administration of PPTF, fasting blood glucose was significantly reduced, oral glucose tolerance and insulin tolerance were markedly improved, diabetes-induced weight loss was alleviated, and hepatic and muscle glycogen contents were restored. Serum TC, TG, and LDL-C levels were decreased, while HDL-C levels were increased. Histopathological damage such as hepatic steatosis and islet compensatory hypertrophy is mitigated to various extents. In summary, an efficient extraction and purification process for PPTF was established. PPTF exhibited favorable hypoglycemic and lipid-regulating effects in T2DM mice, along with potent antioxidant and α-glucosidase inhibitory activities in vitro. These findings point to a multi-faceted mode of action that merits further investigation, although the involvement of key insulin signaling molecules was not examined in the present study. Additionally, approximately 30% of the components in the purified PPTF fraction remain unidentified and warrant further investigation. Despite these limitations, the present findings provide a solid foundation for the further development of this traditional Chinese medicinal resource as a candidate adjuvant functional product for glycemic management.

## Figures and Tables

**Table 1:** Box–Behnken design with 17 experimental runs and observed response values.

No.	Ethanol Volume Fraction (%)	Ionic Liquid Concentration (g/L)	Ultrasonic Power (W)	Extraction Yield (mg/g)
1	70	8	300	37.8
2	80	6	300	36
3	90	8	150	37.6
4	80	8	225	39.7
5	90	6	225	37.2
6	90	8	300	37.3
7	70	10	225	38
8	80	8	225	39.7
9	70	8	150	37.5
10	80	10	150	37.3
11	80	8	225	39.9
12	80	10	300	36.3
13	80	8	225	39.6
14	70	6	225	36.7
15	80	6	150	35.9
16	80	8	225	39.6
17	90	10	225	37.3

**Table 2:** Analysis of variance for the regression model.

Source	Sum of Squares	DF	Mean Square	F-Value	*p*-Value	Significance
Model	29.48	9	3.28	129.2	<0.0001	***
A—Ethanol volume fraction	0.0450	1	0.0450	17.7	0.2245	n.s.
B—Ionic liquid concentration	1.20	1	1.20	47.37	0.0002	**
C—Ultrasonic power	0.1013	1	0.1013	3.99	0.0858	n.s.
AB	0.3600	1	0.3600	14.2	0.007	**
AC	0.0900	1	0.0900	3.55	0.1016	n.s.
BC	0.3025	1	0.3025	11.93	0.0106	*
A^2^	1.58	1	1.58	62.29	<0.0001	***
B^2^	13.45	1	13.45	530.55	<0.0001	***
C^2^	9.95	1	9.95	392.52	<0.0001	***
Residual	0.1775	7	0.0254			
Lack of fit	0.1175	3	0.0392	2.61	0.1883	n.s.
Pure Error	0.0600	4	0.0150			
Cor Total	29.66	16				

R^2^ = 0.994, Adj-R^2^ = 0.986, Pred-R^2^ = 0.972, Adeq Precision = 28.5, CV% = 1.02. *** p<0.001, ** p<0.01, * p<0.05, n.s. = not significant.

**Table 3:** Parameters of HPLC method validation results.

Components	Repeatability (μg, Mean ± SD)	RSD	Stability (μg, Mean ± SD)	RSD	Sample Recovery (%, Mean ± SD)	RSD
Hyperoside	341.30 ± 2.11	1.34%	342.00 ± 7.52	1.42%	98.59 ± 0.58	0.37%
Kaempferol-3-O-rutinoside	165.59 ± 2.11	1.61%	164.30 ± 2.12	1.13%	99.23 ± 1.38	0.88%
Quercetin	250.10 ± 5.83	1.27%	251.10 ± 1.96	0.94%	98.72 ± 0.95	0.83%

**Table 4:** Contents of the three flavonoids before and after purification.

Components	Content After Purification (μg/mL)	Content Before Purification (μg/mL)
Hyperoside	1501.08	593.29
Kaempferol-3-O-rutinoside	77.51	26.86
Quercetin	51.75	20.81

**Table 5:** Box–Behnken design experimental factors and levels table.

Factor	Level Code		
	–1 (Low)	0 (Medium)	+1 (High)
A: Ethanol volume fraction (%)	70	80	90
B: Ionic liquid concentration (g/L)	6	8	10
C: Ultrasonic power (W)	150	225	300

## Data Availability

No new data were created or analyzed in this study. Data sharing is not applicable to this article.
